# Size and Surface Effects in the Ultrafast Dynamics of Strongly Cooperative Spin‐Crossover Nanoparticles

**DOI:** 10.1002/smll.202405571

**Published:** 2024-11-10

**Authors:** Tyler N. Haddock, Teresa Delgado, Marc Alías‐Rodríguez, Coen de de Graaf, Cristian Enachescu, Renske M. van der Veen

**Affiliations:** ^1^ Department of Chemistry University of Illinois Urbana‐Champaign Urbana IL 61801 USA; ^2^ Chimie ParisTech‐CNRS IRCP (PSL) 11 Rue P. et M. Curie Paris 75005 France; ^3^ Departament de Química Física i Inorgànica Universitat Rovira i Virgili Marcel·lí Domingo 1 Tarragona 43 007 Spain; ^4^ Department of Physics Alexandru Ioan Cuza University Blvd. Carol I, nr. 11 Iasi 700506 Romania; ^5^ Department for Atomic‐Scale Dynamics in Light‐Energy Conversion Helmholtz Zentrum Berlin für Materialien und Energie GmbH Hahn‐Meitner‐Platz 1 14109 Berlin Germany; ^6^ Institute of Optics and Atomic Physics Technical University of Berlin Straße des 17. Juni 135 10623 Berlin Germany

**Keywords:** femtosecond optical spectroscopy, photoswitching dynamics, size reduction effects, spin‐crossover nanoparticles

## Abstract

Cooperative photoinduced switching of molecular materials at the nanoscale is still in its infancy. Strongly cooperative spin‐crossover nanomaterials are arguably the best prototypes of photomagnetic and volume‐changing materials that can be manipulated by short pulses of light. Open questions remain regarding their non‐equilibrium dynamics upon light excitation and the role of cooperative elastic interactions in nanoscale systems that are characterized by large surface/volume ratios. Femtosecond‐resolved broadband spectroscopy is performed on nanorods of the strongly cooperative Fe‐triazole, which undergoes a reversible low‐spin to high‐spin (HS) phase transition ≈360 K. Supported by density functional theory and mechano‐elastic Monte Carlo simulations, a marked difference is observed in the photoswitching dynamics at the surface of the nanoparticles compared with the core. Surprisingly, under low excitation (<2%) conditions, there occurs a transient increase in the HS population at the surface on the picosecond time scale, while the HS population in the core decays concomitantly. These results shed light onto the importance of surface properties and dynamical size limits of nanoscale photoresponsive nanomaterials that can be used in a broad range of applications.

## Graphical abstract

An ultrafast spectroscopy study on spin‐crossover nanoparticles displays different dynamics between surface and core states. Due to relaxed pressure at the surface, upon photoexcitation, a 30‐picosecond amplification of high‐spin population occurs at the surface, while core high‐spin population decays. This reveals the importance of surface properties and size limits of photoresponsive nanomaterials which are useful in varied applications.

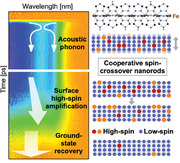

## 1 Introduction

One of the scientists’ long‐standing dreams is the deliberate, reversible control and tunability of material properties such as color, conductivity, magnetization, and structure. In view of applications in optoelectronics, spintronics, and high‐density data storage, many studies are geared toward miniaturization of multifunctional materials in the form of nanostructures and manipulation (“switching”) by using short pulses of laser light. Photoinduced phase transitions, that is, the light‐driven macroscopic transformation of a material from one stable phase into another (meta)stable phase with different electronic and/or structural orders, constitute a particularly attractive method and have received much attention in various types of solids such as organic charge‐transfer compounds, metal oxides, inorganic complexes, and metals.^[^ ^1^ ^]^

Spin‐crossover (SCO) compounds are prototype molecular photomagnetic and volume‐changing materials.^[^ ^2^ ^]^ The process of SCO involves the rearrangement of electrons in the bonding and anti‐bonding valence d‐orbital manifolds of 3d^4^‐3d^7^ transition metal atoms enabling the reversible switching between the low‐spin (LS) and high‐spin (HS) states upon the application of an external perturbation such as temperature, pressure, magnetic field, light irradiation, or absorption of guest molecules.^[^ ^3^, ^4^, ^5^ ^]^ The population of anti‐bonding orbitals in the LS→HS SCO process imparts a sizeable increase in the metal–ligand bond length, which gives rise to reinforcing (“cooperative”) short‐ and long‐range elastic interactions between the spin‐state centers in the solid phase. After light irradiation, locally switched HS states modulate the pressure on surrounding LS sites which, for a certain density of excited states, causes a positive feedback process and a cascade (“molecular domino”) of LS→HS switching events in the near vicinity of the photoexcited HS center.^[^ ^6^, ^7^, ^8^, ^9^, ^10^, ^11^, ^12^, ^13^, ^14^, ^15^ ^]^ This cooperative “elastic amplification”—in which one absorbed photon triggers the transformation of several molecules—should enable fast and highly efficient photoswitching with rather low laser excitation fluences, offering exciting applications in many fields.^[^ ^16^, ^17^, ^18^, ^19^ ^]^

However, the importance of long‐range elastic interactions on SCO material properties poses the question: To what extent can cooperativity be maintained at the nanoscale? Previous experimental and theoretical work has primarily focused on the effect of size reduction on the steady‐state thermal properties such as the increase of the residual HS fraction at low temperatures, the decrease of the spin transition temperature, and the narrowing of the thermal hysteresis width of the phase transition, leading to an ineluctable loss of the LS↔HS bistable phenomenon.^[^ ^20^, ^21^, ^22^, ^23^, ^24^, ^25^ ^]^ A remaining key issue in the field concerns the dynamic, non‐equilibrium evolution of spin‐state switching in cooperative SCO materials at the nanoscale.^[^ ^6^, ^12^, ^26^ ^]^

The ultrafast photophysics of SCO molecules in solution has been extensively studied with time‐resolved optical^[^ ^27^, ^28^, ^29^, ^30^ ^]^ and X‐ray spectroscopies.^[^ ^31^, ^32^, ^33^, ^34^ ^]^ These results have generally shown a sub‐picosecond (ps) intersystem crossing from the singlet (charge‐transfer or metal‐centered) photoexcited states to the HS manifold with near‐unity quantum yield.^[^ ^35^, ^36^ ^]^ Ultrafast studies for SCO solids have been reported for complexes doped in an inert lattice,^[^ ^28^ ^]^ microcrystals embedded in a polymer matrix,^[^ ^37^, ^38^ ^]^ and single crystals.^[^ ^39^, ^40^, ^41^, ^42^ ^]^ Only a handful of ultrafast studies on SCO nanoparticles have been performed.^[^ ^37^, ^43^, ^44^, ^45^, ^46^, ^47^ ^]^ Open questions remain:^[^ ^12^, ^26^, ^48^, ^49^, ^50^ ^]^ How do the kinetics and efficiencies of local switching in SCO nanoparticles depend on their size? How do elastic effects vary between bulk and surface geometries? What role does thermal energy play in HS “amplification”?

Here, we report on a broadband femtosecond (fs) optical transient absorption (OTA) spectroscopy study of the photoinduced dynamics in strongly cooperative [Fe(Htrz)_2_(trz)](BF_4_) (Fe‐trz; trz = 1,2,4‐triazolato; Htrz = 1,2,4‐H‐triazole) SCO nanoparticles. Fe‐trz belongs to the class of 1D coordination polymers.^[^ ^51^, ^52^, ^53^, ^54^ ^]^ It exhibits remarkable properties such as unusually strong cooperativity which subsists at the nanoscale^[^ ^22^, ^55^, ^56^, ^57^, ^58^ ^]^ and high‐energy metal‐to‐ligand charge‐transfer (MLCT) states that can be excited in the UV.^[^ ^28^, ^59^, ^60^, ^61^ ^]^ Nanorods of Fe‐trz display a thermal LS↔HS phase transition at 360 K with a hysteresis width of 10–40 K.^[^ ^22^, ^55^, ^56^, ^57^, ^58^ ^]^ In this study, we conduct fs‐resolved spectroscopy experiments in a low‐excitation regime, where the initially photoexcited HS population does not exceed 2%. Previous studies on solids and nanoparticles have shown that such low excitation levels are below the threshold for elastic amplification of the HS population.^[^ ^6^ ^]^ We observe size‐dependent coherent oscillations (ps) in the UV–vis OTA spectrum resulting from acoustic breathing (radial) expansion of the nanoparticles. By performing a global target analysis of the broadband 2D (wavelength‐time) spectroscopy data, we are able to kinetically and spectrally resolve the dynamics of spin‐state centers located at the surface and in the core of the nanoparticles. From this analysis, we observe that in the ≈35 ps following excitation, there is an amplification of HS centers at the surface, while HS centers in the *core* decay monotonically. Non‐equilibrium mechano‐elastic Monte Carlo (MC) simulations based on the ball‐and‐spring model^[^ ^62^, ^63^ ^]^ complement our results and show that the nanoscale HS evolution results from an intricate interplay between local transient pressure and temperature gradients that are different at the surface and in the core. Using density functional theory (DFT), we show that coordination defects and reduction in ligand‐field symmetry of the Fe centers at the surface of the nanoparticles stabilize the HS state and increase the oscillator strength of metal‐centered transitions in the UV–vis spectrum. The assignment is corroborated by studying the dependence on nanoparticle size. The weight of ΔHS for Fe centers at the surface of the particles increases with decreasing particle size.

Our results demonstrate that cooperative switching dynamics can vary greatly between surface and core geometries in SCO nanoparticles. Surface states are generally described as lower in cooperativity,^[^ ^64^ ^]^ yet due to lower external pressure, we find they favor transient LS→HS conversion. As such, we believe our results have important implications for the dynamical limits of photoswitching in volume‐changing molecular nanomaterials and the control of non‐volatile information using light in general.

## 2 Results and Discussion

### 2.1 Steady‐State Characterization

**Figure 1:**
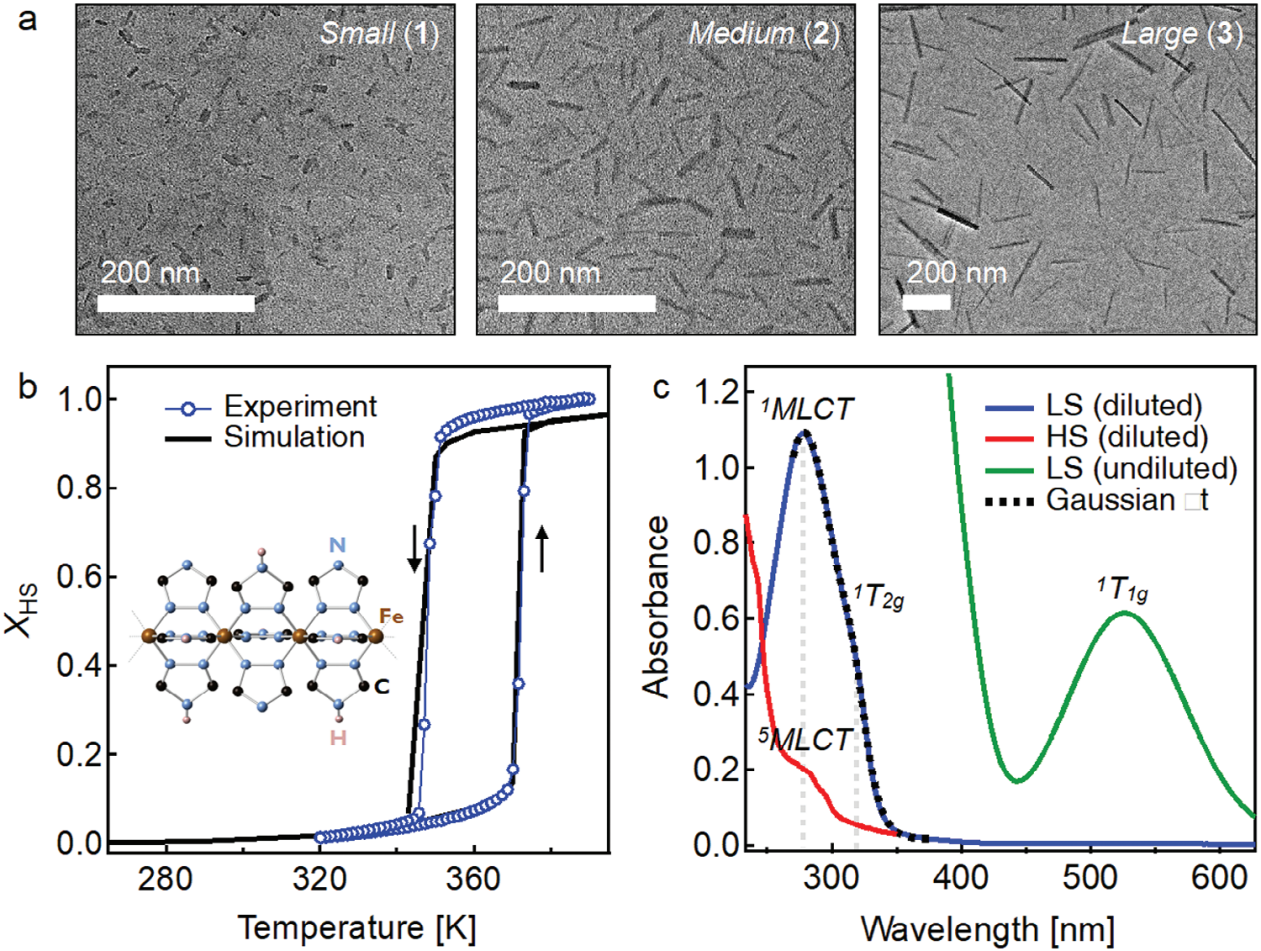
a) TEM micrographs of Fe‐trz nanoparticles. b) Phase transition curve of medium‐sized particles (**2**) measured by SQUID) (blue). A curve generated from mechano‐elastic simulations with a spring constant of 11 N m^−1^ is shown in black. The experimental curve is scaled to compare with the simulation (see Section S7.2, Supporting Information). The arrows indicate the heating (up) and cooling (down) branches. The molecular structure of Fe‐trz is shown as an inset. c) UV–vis absorption spectrum of unwashed Fe‐trz nanoparticles (medium) in octane. Green and blue traces: LS spectrum at room temperature at different concentrations. Red trace: HS spectrum at 390 K (same concentration as blue curve). The relevant electronic transitions are labeled with grey vertical lines. A 2‐Gaussian spectral fit in the UV region is shown as a dashed black line.

Fe‐trz nanoparticles were prepared using the reverse‐micelle technique reported previously.^[^ ^22^, ^55^, ^56^, ^57^ ^]^ The synthesis was adapted to achieve size variability while maintaining a stable dispersion for spectroscopic study. Washing the particles in ethanol with centrifugation removed most of the surfactant (Figure S48, Supporting Information). The nanoparticles could then be resuspended into acetonitrile and remain stable. A detailed description of our synthetic methods is given in Figure S1 (Supporting Information). We synthesized three sizes of Fe‐trz nanoparticles: small (14 × 4.5 × 4.5 nm^3^, **1**), medium (39 × 6 × 6 nm^3^, **2**), and large (150 × 15 × 15 nm^3^, **3**). The small particles (**1**) are to our knowledge the smallest reported for the nanorod morphology. Representative transmission electron microscope (TEM) images are shown in **Figure** **1**a (see Section S2.1, Supporting Information for analysis of size distribution). An additional batch of medium particles (39 × 6 × 6 nm^3^, 2*) was synthesized to test the batch‐to‐batch variability (see Figure S2, Supporting Information for characterization).

Figure 1b shows the thermal phase transition curve obtained from SQUID (superconducting quantum interference device) and the molecular structure of Fe‐trz.^[^ ^65^ ^]^ The phase transition properties are in agreement with previous Fe‐trz nanoparticle magnetometry measurements.^[^ ^55^, ^56^, ^66^, ^67^ ^]^ Fe‐trz is composed of [Fe(Htrz)_2_(trz)]*_n_* chains^[^ ^65^ ^]^ with significant intra‐ and interchain interactions that are responsible for its strong cooperativity.^[^ ^68^ ^]^ The long axis of the nanorods is aligned along the [Fe(Htrz)_2_(trz)]*_n_* chain direction.^[^ ^66^, ^69^ ^]^ The coherent‐domain size was reported to be 10–50 nm.^[^ ^65^ ^]^ This means that the small and medium‐sized particles are assumed to be single crystalline. Even with as few as ≈1000 Fe unit cells (small‐sized particles, **1**), the particles still show a thermal hysteresis width of 25 K. The phase transition temperatures and the square (sharp) hysteresis shape are consistent with polymorph I (Section S2.2, Supporting Information), but we cannot discount the presence of some polymorph II.^[^ ^53^ ^]^

The UV–vis absorption spectrum (Figure 1c) measured at room temperature in acetonitrile shows intense transitions in the UV. Hereafter, we will typically reference the LS and HS states using their Mulliken symbols, namely, the ^1^A_1_ _g_ and ^5^T_2_ _g_ states, respectively. The strongest UV–vis absorption feature is due to an MLCT band (^1^A_1g_→^1^MLCT) at ≈280 nm. The magnitude of this transition is significantly weakened in the HS phase (^5^T_2g_→^5^MLCT) due to the longer metal–ligand bond. The shoulder observed ≈320 nm is due to the ^1^A_1g_→^1^T_2g_ metal‐centered d–d transition. This formally Laporte‐forbidden transition is much more intense than the lowest‐energy ^1^A_1g_→^1^T_1g_ d–d transition in the visible region ≈520 nm.^[^ ^60^ ^]^ This is likely a result of intensity borrowing from the intense ^1^MLCT band nearby.^[^ ^70^ ^]^ The UV–vis absorption spectra for all washed particles are shown in Figures S6–S9 (Supporting Information). The spectral overlap of the broad MLCT and d–d bands in the UV prevents observation of any electronic state perturbation due to size effects.

### 2.2 Femtosecond Transient Absorption Spectroscopy

**Figure 2:**
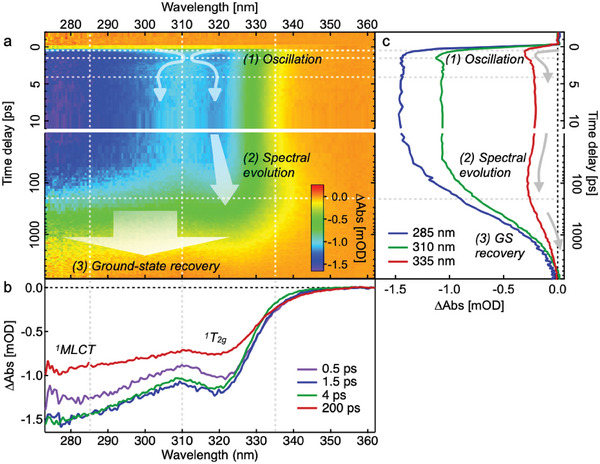
OTA data for Fe‐trz nanoparticles (**2**) with excitation at 267 nm. a) 2D wavelength‐time spectrum. The three main dynamical features are indicated by arrows. Note the partial logarithmic scale on the time‐delay axis. b) Transient spectra at different time delays which are denoted as vertical dashed lines in (a). c) Kinetic traces at different wavelengths which are denoted as horizontal dashed lines in (a).

Ultrafast pump‐probe OTA spectroscopy was performed on all three sizes of washed Fe‐trz nanoparticles after excitation at 267 nm into the ^1^MLCT band at 288 K (see Figure S3, Supporting Information for experimental details and more data). **Figure** **2**a shows the 2D wavelength‐time plot for the medium‐sized particles (**2**). The spectra have been corrected for an artifact originating from the photoexcited solvent (see Section S3.2, Supporting Information for details). The transient spectra in Figure 2b show the prompt appearance of two negative peaks at ≈280 and 320 nm corresponding to ground state bleach (GSB) signals of the ^1^A_1g_→^1^MLCT and ^1^A_1g_→^1^T_2g_ transitions, respectively. Negative GSB signals reflect a depleted ground state absorption due to the transition to higher‐lying excited states upon photoexcitation. The GSB signal that is nearly a mirror image of the ground state absorption spectrum (Figure 1c) indicates the formation of a transient species within the instrumental response time of ≈200 fs which does not absorb significantly in this wavelength range. These results are consistent with the ultrafast population of the ^5^T_2g_ (HS) state upon ^1^MLCT excitation as seen in other Fe SCO systems^[^ ^27^, ^28^, ^34^, ^37^, ^41^, ^71^ ^]^ (see Figure S4, Supporting Information for confirmation by ultrafast extreme UV (XUV) spectroscopy experiments on Fe‐trz nanoparticle deposited thin films). We do not observe any intermediate states between the ^1^MLCT Franck‐Condon state and the ^5^T_2_ _g_ state within the temporal resolution of the experiment. We estimate that we photoexcite 0.5–1.1% LS→HS centers using an incident laser fluence of <2 mJ cm^−2^ (see Section S3.4, Supporting Information for details). The nanoparticles are homogeneously excited because the optical laser penetration depth (>200 µm) is much larger than the nanoparticle size (<150 nm).

The OTA data of all three nanoparticle sizes are characterized by three distinct processes occurring on different time scales, indicated in Figure 2: 1) a few‐ps oscillation, 2) a spectral evolution (broadening/shift) of the ^1^A_1g_→^1^T_2g_ GSB feature on the time scale of tens of ps, and 3) a ^5^T_2g_→^1^A_1g_ ground state recovery on the ≈1 nanosecond (ns) time scale. We will first discuss the size‐dependent oscillation feature (1). We will then analyze the ground state recovery (3) in more detail, after which we will discuss the origin of the spectral evolution (2).

#### 2.2.1 Acoustic Wave Oscillation

During the first few ps after excitation, a clear modulation of GSB intensity is seen in the wavelength range of 300–325 nm (Figure 2). Inspection of the spectral traces taken at different time delays (Figure 2b) shows that the modulation mainly involves a change in ^1^A_1g_→^1^T_2g_ GSB intensity, with a slight concurrent blue shift of this band during the first ps. The oscillation period ranges from 2 to 15 ps depending on the particle size. We thus assign these oscillations to coherent acoustic phonons of the nanoparticles, the time scale of which is dictated by the speed of sound in the material and the nanoparticle size as detailed below.

Acoustic phonon oscillations of metallic nanoparticles have been extensively studied in the past.^[^ ^72^, ^73^, ^74^ ^]^ In SCO materials, they have been observed in molecular crystals.^[^ ^39^, ^42^, ^75^, ^76^ ^]^ While in metallic systems the thermal expansion is solely responsible for the launching of acoustic phonons, in SCO materials, the instantaneous (<200 fs) creation of HS centers upon photoexcitation causes the lattice to expand. The increased volume of the HS molecules (in addition to thermal expansion) causes elastic strain in the lattice which leads to the excitation of acoustic waves affecting the entire nanoparticle. The spectral signature of the oscillation does not arise from any spin state change.^[^ ^32^ ^]^ The intensity changes are associated with the modulation of the MLCT band energy due to the structural distortion during oscillation.^[^ ^42^ ^]^ In the following, we will analyze the size dependence of the oscillation period and type of acoustic phonon mode.

**Figure 3:**
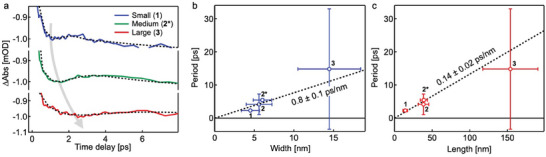
a) Kinetic traces at 305 nm for **1**, 2*, and **3**. The dashed lines show the results from a global fit (see Figure S5, Supporting Information). b,c) The dependence of the acoustic phonon period for **1**, **2**, 2*, and **3** as a function of nanoparticle width (b) and length (c). The dashed lines show linear fits through the origin.

Representative kinetic traces at 305 nm for all three nanoparticle sizes are shown in **Figure** **3**a. Due to low signal‐to‐noise levels in the low‐excitation regime (<2 mJ cm^−2^; ≈1% ΔHS fraction), we acquired data at higher excitation levels (≈12 mJ cm^−2^; ≈10% HS fraction) to analyze the oscillation feature. In Figure S25 (Supporting Information), we show that the amplitude of the oscillation scales linearly with excitation density, while the frequency remains the same. In the high‐power data, an amplification of the signal at later times (tens of ps) is observed which does not appear in the low‐power data. This amplification will be the subject of a future publication. Figure 3a shows the results of fits on kinetic traces at 305 nm with a function including a damped cosine, an instrument‐response limited step‐jump representing the LS→HS excitation, and an additional ≈10′s of ps rise to reflect the amplification step. Details of the fitting are given in Sections S5.2,S5.3 (Supporting Information). In Figure 3b,c, we present the fitted oscillation periods as a function of nanoparticle width and length. The error bars are the standard deviations of the particle dimensions (from TEM analysis, see Table S1, Supporting Information) and the standard deviations of the period parameter in the fit (Table S5, Supporting Information). The increased error bars for the large particles are due to the relatively shorter damping time. The large particles’ oscillations dampen in fewer cycles, leading to greater uncertainty in the fit. The size dependences were fit with a line passing through zero and weighted by the abscissa and ordinate error bars. A slope of 0.8 ± 0.1 ps nm^−1^ was obtained for the width dependence, and a slope of 0.14 ± 0.02 ps nm^−1^ was obtained for the length dependence. In order to test whether the acoustic phonons are associated with longitudinal or transverse motions, we analyze both hypotheses in the following paragraph.

If we approximate our nanorod geometry as a cylinder with isotropic elasticity, the frequency of the acoustic standing waves can be predicted from the particle length, *L*, and diameter, *d*.^[^ ^77^ ^]^ In the limit where *L* is much larger than *d*, two types of acoustic modes are present: extensional modes, that is, vibrations along the length of the nanorods (period *T*_ext_), and breathing modes, that is, vibrations along their radius (period *T*_br_). To assign our oscillation to one of these fundamental modes, we consider the dependence of the oscillation period on the speed of sound *c* in the material along with the cylinder dimensions:

(1) $$T_{\mathrm{ext}}=\frac{2L}{c}\;$$

(2) $$T_{\mathrm{br}}=\frac{\pi d}{\tau_{1}c}\;\sqrt{\frac{\left(1+\nu \right)\left(1-2\nu \right)}{\left(1-\nu \right)}}$$

where *ν* is the Poisson ratio, and *τ*_1_ is the first root of the equation *τJ*_0_(*τ*) = [(1 – 2*ν*)/(1 − *ν*)]*J*_1_(*τ*), where *J_m_* values are the Bessel functions of the first kind. The Poisson ratio for Fe‐trz is not known a priori. Based on the Poisson ratio of 0.34 ± 0.04 of a similar SCO material ([Fe(HB(trz)_3_)_2_]),^[^ ^78^ ^]^ we use a conservative range (*ν* = 0.25–0.45) to make an order‐of‐magnitude estimate for the breathing‐mode speed of sound. Using the fitted slopes from Figure 3b,c, we calculate *c*_ext_ = 15 000 ± 2000 m s^−1^ for the extensional mode hypothesis and *c*_br_ = 1000–2000 m s^−1^ for the breathing mode hypothesis (see Section S5.4, Supporting Information for details). The sound velocity for [Fe(HB(trz)_3_)_2_] was estimated at 1810 ± 45 m s^−1^.^[^ ^78^ ^]^ Likewise, other SCO materials are reported to have sound velocities between 2000 and 4000 m s^−1^.^[^ ^14^, ^15^, ^42^, ^79^, ^80^ ^]^ The 15 000 m s^−1^ velocity derived for an extensional mode is unphysical and thus we reject this hypothesis. Therefore, we assign the oscillation we see in our data as an acoustic breathing mode. Indeed, this mode is predicted to be the strongest frequency component for cylindrical particles.^[^ ^77^ ^]^ In a recent study on Fe‐trz which directly probed the structural distortion using X‐ray scattering, both transverse and longitudinal strain waves were observed with comparable speeds estimated at ≈2.5–3.8 km s^−1^.^[^ ^81^ ^]^ One reason we do not also observe the lower frequency extensional mode is because of its expected short damping time due to the size dispersity of the nanoparticles (Table S7, Supporting Information).

The size‐dependent acoustic phonons revealed here demonstrate the ultrafast (≈ps) radial expansion of the nanoparticle lattice due to the initial photoexcitation of HS centers in the lattice. Such fast expansion waves have been proposed to trigger a subsequent increase of the fraction of HS molecules at longer times.^[^ ^6^, ^11^, ^13^, ^26^, ^62^ ^]^

#### 2.2.2 High‐Spin Decay

**Figure 4:**
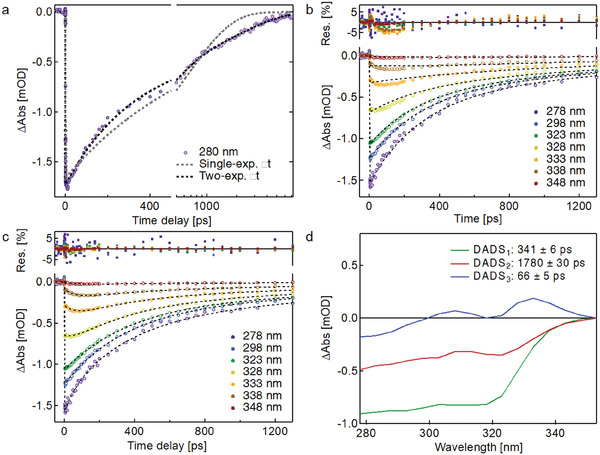
a) Single‐ and two‐exponential fits to the kinetic trace at 280 nm (medium‐sized particles **2**). b) Representative kinetic traces with their fits (dashed lines) from a two‐exponential global analysis. The fit residuals are shown on the top of the graph. c) Same as in (b), but for a three‐exponential global analysis. d) DADS obtained from the three‐exponential global analysis.

An initial assessment of the dynamics of ground state recovery can be made by fitting single‐wavelength kinetic traces of the GSB signal versus time. Since the HS ^5^T_2g_ state is the lowest energy excited state,^[^ ^82^ ^]^ the GSB recovery directly reflects the HS→LS back‐relaxation process. **Figure** **4**a shows mono‐ and biexponential fits to a kinetic trace at 280 nm for the medium‐sized particles (**2**) (same data as in Figure 2). Clearly, a single‐exponential function is insufficient to fit the kinetic trace, while a two‐exponential fit with time constants ≈300 ps and ≈2 ns reproduces the GSB recovery very well. The decay times are similar to those measured in diluted SCO crystals and SCO molecules in solution which have high phase transition temperatures like Fe‐trz.^[^ ^83^, ^84^ ^]^ These first fitting results indicate the presence of two distinct HS species that decay on different time scales (vide infra for assignment).

##### Global Analysis

In order to obtain a more holistic and quantitative view on the dynamics, we performed a global analysis using a two‐exponential decay function in the wavelength range 280–355 nm. The results of the global analysis are shown in Figure 4b for a representative set of kinetic traces. It is immediately evident that a third component is necessary to adequately reproduce the dynamics in the range 305–340 nm.

The results for a three‐exponential global analysis of the same data are shown in Figure 4c. The quality of the fit has improved significantly (see Figures S35–S38, Supporting Information). Using the locally fitted amplitudes, we constructed decay‐associated difference spectra (DADS), shown in Figure 4d. A DADS represents the spectral contribution to the 2D transient data set that exponentially decays with a specific time constant. It is constructed from the locally fitted amplitudes of each exponential decay in the overall fitting function (see Sections S6.1,S6.2, Supporting Information). While the first two components, DADS_1_ (341 ± 6 ps) and DADS_2_ (1780 ± 30 ps), have clear ^1^A_1g_→^1^MLCT and ^1^A_1g_→^1^T_2g_ bleach features, the sign and shape of the third decay component DADS_3_ (66 ± 5 ps) looks completely different. DADS_3_ cannot represent a new excited state species based on ultrafast XUV spectroscopy (Figure S4, Supporting Information) and the literature.^[^ ^84^, ^85^ ^]^ Its two‐lobe shape is centered around the ^1^A_1g_→^1^T_2g_ transition ≈320 nm, with a slightly larger positive lobe on the lower energy side. The decay of DADS_3_ therefore represents a broadening of the ^1^A_1g_→^1^T_2g_ bleach band and a shift of spectral weight to longer wavelengths. These dynamics represent the spectral evolution marked as (2) in Figure 2. We note that the GSB ^1^A_1g_→^1^T_2g_ band in DADS_2_ is slightly broadened and red‐shifted compared to DADS_1_. Based on these results, we conclude that the HS population seems to shift from a short‐lived species with a ≈350 ps lifetime to a long‐lived species with an ≈1800 ps lifetime and a broadened and red‐shifted ^1^T_2g_ absorption band. The time scale for this shift is represented by the decay of DADS_3_ within ≈60 ps. We note that these fitted decay times do not show a clear dependence on nanoparticle size (see Section S6.2, Supporting Information).

Non‐Equilibrium Mechano‐Elastic Simulations

The above analysis has shown that there are two HS species that decay back to the LS state on two different time scales (≈340 ps and ≈1.7 ns). In order to identify the nature of these two species, we performed non‐equilibrium mechano‐elastic MC simulations including heat diffusion.^[^ ^62^, ^63^ ^]^ Details of the simulations are given in Section S7.1 (Supporting Information). Briefly, the Fe‐trz centers (LS, HS) in this model are represented as rigid spheres with different radii (*r*_LS_ < *r*_HS_) arranged in a 2D rectangular open boundary lattice in a triangular configuration. The simulations were done for a rod‐shaped lattice composed of 10 368 Fe centers with a length/width ratio equal to 4, which matches roughly the numbers of Fe centers and aspect ratios of the Fe‐trz nanoparticles **1**–**3** (1200−58 000 and 3.2–4.2, respectively). The elastic interactions between spin centers are represented by springs, whose elastic spring constant modulates the cooperativity of the system. The volume change of an Fe center upon spin‐state switching results in an elongation or compression of its closest springs, exerting a local pressure, which will in turn determine the change in position of neighboring molecules and a propagation of the initial perturbation through the entire lattice. At time zero (MC step, MCS = 1), a homogeneous photoexcitation is emulated by randomly choosing a fraction of LS to HS conversions and increasing their temperature to *T* = 850 K. This high initial temperature results from the large excess energy (4.38 eV) put into the molecule by photoexciting in the UV (*hν* = 4.66 eV, compared to the HS state energy of 0.28 eV^[^ ^53^ ^]^). The excess energy is released into the surroundings in the form of heat. The evolution of the system during the relaxation is determined by MC Arrhenius dynamics.^[^ ^86^, ^87^ ^]^ At each MCS, the switching probabilities for all Fe centers in the system are computed and they are compared with random numbers generated in the range [0, 1]. If the transition probability is higher than the random number, the switching is accepted and the molecule flips to the new state; otherwise, it keeps its previous state. In each MCS, the new positions of all Fe centers are computed and their new temperatures are calculated according to a heat diffusion model with two thermal bath temperatures (one internal and one external, see Section S7.3, Supporting Information).^[^ ^63^ ^]^

Steady‐state MC simulations as a function of temperature were performed to reproduce the thermal phase transition curve in Figure 1b (see Section S7.2, Supporting Information for details). Using Fe‐trz specific parameters for Δ*r* = 0.2 Å, Δ*H* = 3251 K, Δ*S* = 9.06 J K^−1^ (or *g* = 8604), and a phase transition temperature of *T*_c_ = 359 K,^[^ ^53^ ^]^ a spring constant of 11 N m^−1^ was determined from the steady‐state SQUID data (Figure 1b). This spring constant is larger than typical values in the literature, as expected for the unusually strong cooperativity of the polymeric Fe‐trz material.^[^ ^22^, ^65^, ^68^ ^]^ We note that there is a small (0.4%) fraction of HS centers at RT. However, the slope of the SQUID data ≈288 K is small, which means that the equilibrium HS fraction does not significantly change for slight temperature increases (Δ*T* ≈ 30 K) due to photoexcitation. The derived spring constant was then used in the non‐equilibrium MC simulations.

**Figure 5:**
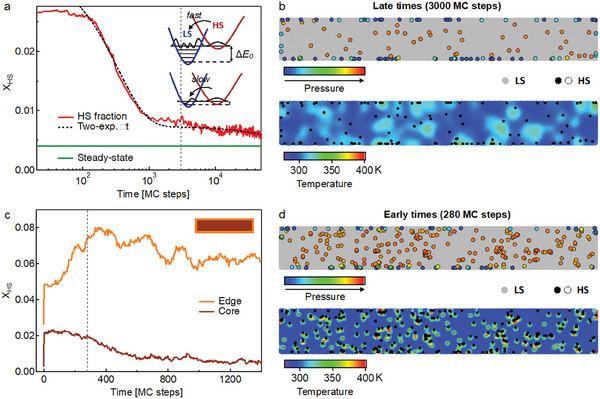
Results from MC simulations. a) Total HS fraction as a function of time (in MCS) together with a two‐exponential fit to the data (dashed black curve, fit starts at MCS = 100). The steady‐state HS fraction at 288 K is indicated by a green line. The inset shows how lowering the zero‐point energy Δ*E*_0_ results in a slower HS→LS back‐relaxation. b) Snapshot from the MC simulation at 3000 MCS, indicated with a vertical dashed line in (a). Top) Pressure variations for HS centers (open circles, arbitrary pressure units). Bottom) Temperature variations of the lattice. HS centers are denoted by closed circles. c) Edge and core HS fraction as a function of time for early time delays. Fractions are relative to the respective number of Fe centers at the edge (887) and in the core (9481). A schematic of the nanorod with edge (orange) and core (brown) areas is shown in the top right. d) Snapshot from the MC simulation at 280 MCS, indicated with a vertical dashed line in (c). Top) Pressure variations for HS centers (open circles, arbitrary pressure units). Bottom) Temperature variations of the lattice. HS centers are denoted by closed circles.

**Figure** **5** shows the results from non‐equilibrium MC simulations for an initial photoexcited HS population of 2%. Several MC simulations were averaged together to improve the statistics (see Figure S7, Supporting Information for details). The long‐time scale dynamics (Figure 5a) show a decay of the HS fraction that can be fitted satisfactorily by a two‐exponential function, in good agreement with the two‐exponential decay of the GSB in the experimental data (Figure 4). A snapshot of the lattice at late times, when the fast decay has largely subsided, shows an accumulation of HS centers close to the edges of the lattice (the edge is defined as comprising the 3 outer layers of Fe centers). At 3000 MCS, the increase in HS fraction at the edge (6%) is larger than that in the core (0.3%), while the initial excitation fraction at time zero was homogeneous across the particle (the steady‐state HS fractions for the edge, 2.5%, and the core, 0.3%, are subtracted). This shows that the HS→LS decay on longer time scales (≈1.7 ns, Figures 2, 4) can be associated with HS centers located close to or on the surface of the nanoparticles. Indeed, Figure 5b (Top) shows that these centers experience a lower local pressure compared to HS states in the center of the particles, which is due to the open boundary of the lattice close to the edge. Lower pressure decreases the ligand field strength, which in turn lowers the zero‐point energy Δ*E*_0_^[^ ^84^, ^88^, ^89^ ^]^ (defined as the difference in energy between the lowest vibronic states of the LS and HS potentials, inset in Figure 5a). HS centers at and close to the surface of the nanoparticle are therefore relatively stabilized, leading to an increased energy activation barrier for back‐relaxation and subsequently longer HS→LS decay times. The most relaxed geometries may be the end‐of‐chain termini which should most strongly favor the HS state. DFT electronic structure calculations on a Fe‐trz molecular analog show the increased stabilization of the HS state at the terminus (see Figure S47, Supporting Information).

In Figure 5c, we plot the simulated early‐time scale dynamics of the edge and core HS fractions separately (fractions relative to the respective number of molecules in the edge and core regions). We can correlate the fast decay in the total HS fraction (edge + core, Figure 5a), with the decay of HS fraction in the core of the nanoparticles. Overall, we can thus assign the slow ≈1.7 ns component of the GSB recovery kinetics (Figures 2, 4) to the back‐relaxation of HS molecules primarily located at or near the surface of the Fe‐trz nanoparticles, and the fast ≈340 ps component to the decay of HS molecules primarily residing in the core of the nanoparticles.

The dependence of the HS→LS decay time on the ligand environment has been reported in the literature for SCO materials in solution^[^ ^90^, ^91^ ^]^ and in solids.^[^ ^84^, ^92^, ^93^, ^94^, ^95^ ^]^ In SCO nanoparticles, the different coordination environment and usually lower ligand field of molecules located at the surface gives rise to an increased residual HS fraction below the phase transition^[^ ^20^, ^21^, ^23^, ^57^ ^]^ and a long‐lived metastable HS population after light irradiation at low temperature.^[^ ^96^ ^]^ In a comparison between crystalline and amorphous SCO nanoparticles, a slower HS→LS relaxation kinetics was attributed to a less‐dense packing and an increased number of defects in the amorphous phase.^[^ ^97^ ^]^ Furthermore, mechano‐elastic MC simulations have shown that nucleation of a new phase (LS, HS) starts from corners or edges of an SCO lattice, and HS centers at the edge exhibit slower HS→LS relaxation times.^[^ ^62^, ^63^, ^96^, ^98^ ^]^

We note that our interpretation of two distinct HS species with exponential decay behavior is an oversimplification. In reality, a spread of local pressures and zero‐point energies is expected that leads to a distribution of decay times.^[^ ^28^, ^99^, ^100^ ^]^ Furthermore, in addition to Fe centers at the surface, there may also be distorted Fe centers inside the particles that contribute to the slow decay, for example, Fe centers at chain termini caused by lattice defects. A fit of the GSB decay with a two‐exponential function had a lower sum of squares of the residuals than functions representing distributions of rate constants (stretched exponential,^[^ ^99^ ^]^ Gaussian distribution,^[^ ^100^ ^]^ log‐normal distribution, see Section S6.3, Supporting Information). We thus believe that the adequate modeling of the HS→LS back‐relaxation with a two‐exponential decay function indicates that the strongly cooperative polymeric structure and nanoparticle morphology of the Fe‐trz material leads to two rather well‐defined species of Fe centers. Mechano‐elastic simulations show that these likely correspond to Fe centers close to the surface, experiencing reduced local pressure, and those in the core of the nanoparticles that experience a bulk‐like environment with increased local pressure.

#### 2.2.3 Spectral Evolution

As seen in the simulated early‐time scale dynamics of the edge and core HS fractions in Figure 5c, surprisingly, the HS fraction at the edge increases during the first 400 MCS, while the fraction in the core concurrently decreases. These intriguing dynamics are the result of counteracting effects of local pressure and temperature variations in the lattice. At early times following photoexcitation, the local temperature around HS molecules is high due to the initial photoinduced temperature jump of Δ*T* = 850 K (Figure 5d, Bottom). At the same time, HS molecules in the core experience a large positive pressure because they are embedded in a predominantly LS lattice (Figure 5d, Top). In terms of HS→LS probability, these two effects are counteracting (Equation S13, Supporting Information). While the high pressure supplies a motive to flip to LS, this tendency is placated by the photoinduced temperature jump which favors maintaining the HS state. However, the dynamics of temperature and pressure are very different. Since at these small excitation fractions (2%) the lattice will remain predominantly in the LS phase, pressure will not decrease significantly immediately following photoexcitation. By contrast, the temperature quickly dissipates to neighboring molecules following photoexcitation. This means that the effect of the positive pressure (favoring LS) will ultimately outweigh the thermal effect (favoring HS). Therefore, the core HS population will tend toward the equilibrium population expected for a slightly elevated average temperature (Section S7.2, Supporting Information).

The situation is different at the surface of the nanoparticles. Figure 5c shows a fast local amplification of HS fraction at the edges, while the total (edge + core) HS population monotonically decreases (Figure 5a). Photoexcited HS states at the edges experience a much lower pressure, given the increased freedom from proximity to the open boundary, which leads to a greater tendency for LS→HS switching. On the other hand, these molecules cool quicker than HS centers in the core since they can efficiently dissipate heat to the adjacent solvent molecules (Section S7.3, Supporting Information).

We note that the average temperature of the nanoparticles (maximum of 305 K) always stays well below the phase transition temperature at ≈360 K (Figure 1b). An appreciable thermal proliferation of the HS population is therefore not expected here, in contrast to previous studies that were conducted at temperatures much closer to the phase transition temperature, which were also more gradual.^[^ ^6^, ^11^, ^12^, ^13^, ^14^, ^26^ ^]^ Locally and on short time scales, however, temperatures can be much higher, well‐exceeding 360 K, as is seen in the MC snapshot at 280 MCS in Figure 5d (bottom). This opens the question whether LS→HS switching can be induced thermally in the near vicinity of hot photoexcited HS molecules and on short (tens of ps) time scales. Such a possibility is included in the simple thermo‐elastic MC model used here (Sections S7.1–S7.3, Supporting Information). However, if we effectively exclude thermal diffusion in the model by setting the thermal diffusion coefficients to exceedingly small values, an accumulation of HS population at the edges is still observed^[^ ^62^ ^]^ (Section S7.4, Supporting Information). Thermal effects thus affect the relative time scales and magnitudes of the various processes, but they are not solely responsible for the overall qualitative observation, namely amplification of HS centers at the surface of the nanoparticles and concurrent decay of HS population in the core. We propose that these HS population dynamics are driven by the tendency toward mechanical equilibrium. After photoexcitation, the lattice expands to a new volume to accommodate the increased HS fraction on a time scale dictated by the speed of sound (few to tens of ps). However, the mechanical equilibrium state at this new (larger) volume would generally prefer HS states at the surface compared to the core, because of the weaker ligand field of molecules at or close to the surface. Therefore, the effective shift of the HS population from the core toward the surface may be thought of as a tendency to reduce the elastic energy and restore mechanical equilibrium transiently. At later times, as the particle volume decreases, the HS population will tend toward the equilibrium HS distribution again (Figure 1b). We note that in this low excitation experiment, HS amplification occurs primarily at the surface of SCO nanoparticles. At much higher excitation densities, the total HS fraction is amplified, which will be the subject of a future publication.

**Figure 6:**
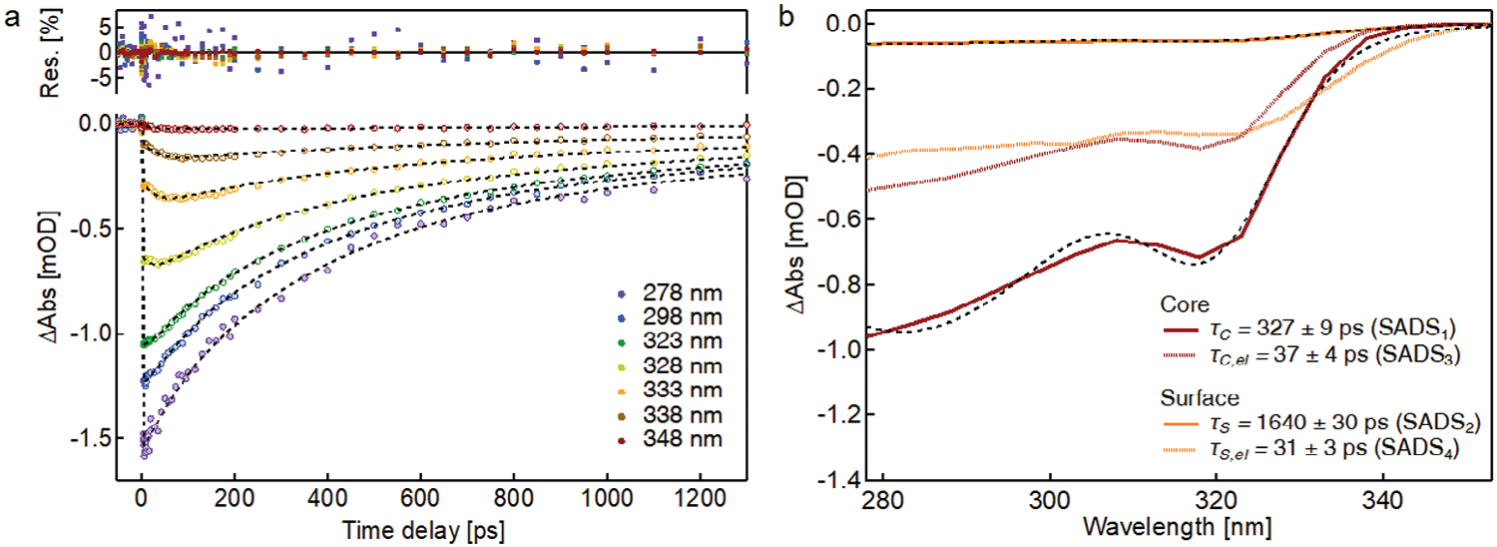
Results from the target analysis of the OTA data. a) Representative kinetic traces with their fits (dashed lines). The fit residuals are shown on the top of the graph. b) Species‐associated difference spectra (SADS based on the locally fitted (weighted) amplitudes *A*_C_ (SADS_1_, decay on time scale *τ*_C_), *A*_S_ (SADS_2_, decay on time scale *τ*_S_)_,_ *x*_C_ *A*_C_ (SADS_3_, decay on time scale *τ*_C,el_) and *x*_S_ *A*_S_ (SADS_4_, *rise* on time scale *τ*_S,el_). The dashed lines are two Gaussian fits.

In order to corroborate this interpretation, we conducted a target fit analysis on the OTA data based on the assignment and dynamics of surface and core HS species discussed above. The fit function (Equation S18, Supporting Information) includes two exponential decay components with amplitudes *A*_C_ and *A*_S_ to represent the back‐relaxation of core (*τ*_C_) and surface (*τ*_S_) HS states on the 0.3–2 ns time scale, respectively. In addition, to model the amplification of surface and (de)amplification of core HS states on faster time scales (Figure 5c), the fit includes an exponential rise function weighted with fraction *x*_S_, and an exponential decay function weighted with fraction *x*_C_, both with independent global time constants, *τ*_S,el_ and *τ*_C,el_, respectively. The amplified surface HS fraction *x*_S_ ultimately decays with time constant *τ*_S_. The results of the target fit for selected wavelengths are shown in **Figure** **6**a. In Table S8 (Supporting Information), we show that considering the smaller number of fit parameters in the target analysis, the fit quality is improved for 3 of the 4 batches compared to the three‐exponential global analysis (Figure 4c). The species‐associated difference spectra (SADS) derived from the fitted (weighted) amplitudes *A*_C_, *A*_S_ *_,_ x*_C_ *A*_C_, and *x*_S_ *A*_S_ are shown in Figure 6b. The spectral shape and decay times of the first two components, SADS_1_ with *τ*_C_ ≈ 330 ps and SADS_2_ with *τ_S_* ≈ 1.6 ns, are similar as obtained from the three‐exponential global analysis (DADS_1_ and DADS_2_ in Figure 4d). The decay (SADS_3_, *τ*_C,el_) and rise (SADS_4_, *τ*_S,el_) of core and surface HS states, respectively, capture the spectral evolution of the GSB ^1^T_2g_ band ≈320 nm (Figure 2), which was previously represented by DADS_3_ in the global analysis (Figure 4d). The ^1^T_2g_ band is slightly red‐shifted and broadened in SADS_4_ compared to SADS_3_, while their amplitudes are similar. The fact that the time scale of HS amplification at the surface (*τ*_S,el_ ≈ 31 ps) and HS (de)amplification in the core (*τ*_C,el_ **≈ 37 ps) are similar corroborates that these processes occur in tandem.

**Figure 7:**
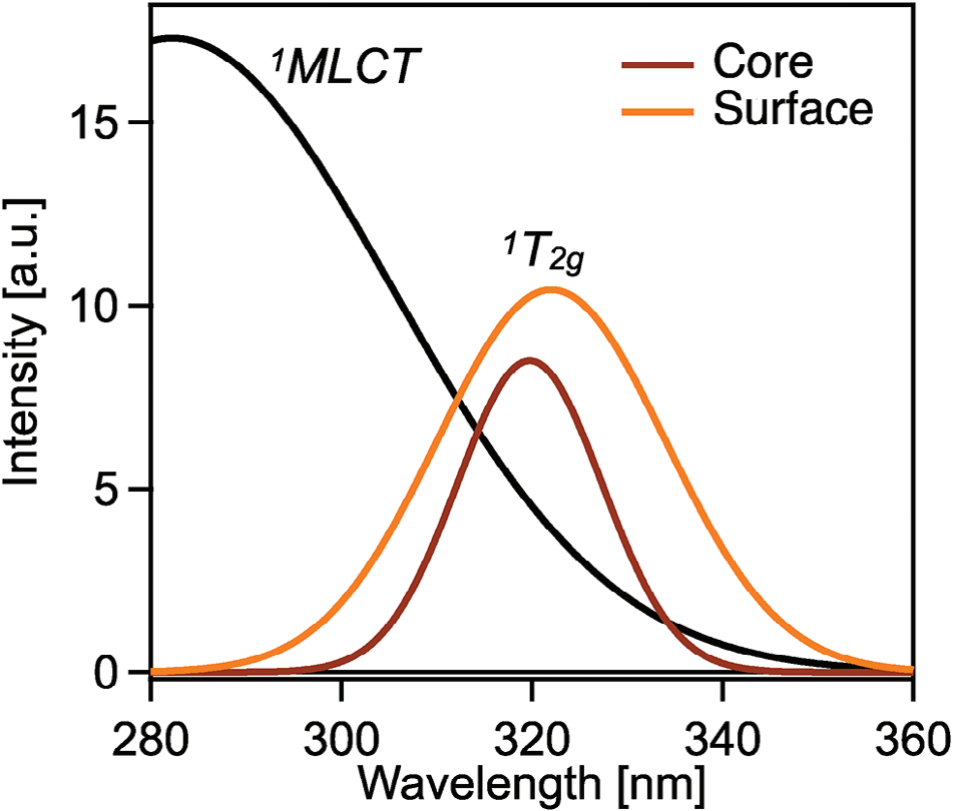
Reconstructed ground state absorption spectra of SCO centers located at the surface and in the core of Fe‐trz nanoparticles obtained from fitting SADS_1_ and SADS_2_ in Figure 6b to a sum of two Gaussians. The width and position of the Gaussian for the ^1^A_1g_→ ^1^MLCT absorption band are held fixed and the spectra are normalized to its area. The ^1^A_1g_→^1^T_2g_ absorption band shifts to longer wavelengths broadens, and its area increases for the SCO centers at the surface.

With the goal of extracting absorption spectra for the surface and core SCO molecules, we fitted SADS_1_ and SADS_2_ each to a sum of two Gaussians, one for the ^1^A_1g_→ MLCT absorption band and one for the ^1^A_1g_→^1^T_2g_ band (see Figure 6b for example fits). The width and position of the MLCT absorption band is held fixed in the fit since we are not able to accurately resolve changes in these parameters because of our limited probing range. The spectra are then normalized to the area of the MLCT absorption band. The results are presented in **Figure** **7** for the medium‐sized particles (**2**) (see Table S9, Supporting Information for all batches). We see that the ^1^A_1g_→^1^T_2g_ absorption band of the surface molecules is shifted to longer wavelengths, is broader, and its area is larger compared to the band of molecules in the core. These spectral changes can be related to the electronic and structural differences between surface and core SCO centers. The red shift is explained by the lower ligand field of the SCO molecules at the surface due to reduced local pressure and therefore ligand‐field strength. As seen in the Tanabe‐Sugano diagram for an octahedral ligand field,^[^ ^101^ ^]^ smaller ligand‐field strength reduces the energy of all states, especially the metal‐centered d–d states such as ^1^T_2g_. The broadening of the band for surface molecules may be due to increased heterogeneity at the surface compared to the core, leading to a range of zero‐point and transition energies. Finally, the increase of oscillator strength of the ^1^A_1g_→^1^T_2g_ absorption band for surface states is corroborated by DFT calculations. In Figure S46 (Supporting Information), we show that for a similar material composed of [Fe(HB(trz)_3_)_2_] molecules, breaking the inversion symmetry of the coordination environment lifts the selection rules for formally forbidden d‐d transitions and therefore sensitively increases the oscillator strength of the ^1^A_1g_→^1^T_2g_ absorption band.

**Figure 8:**
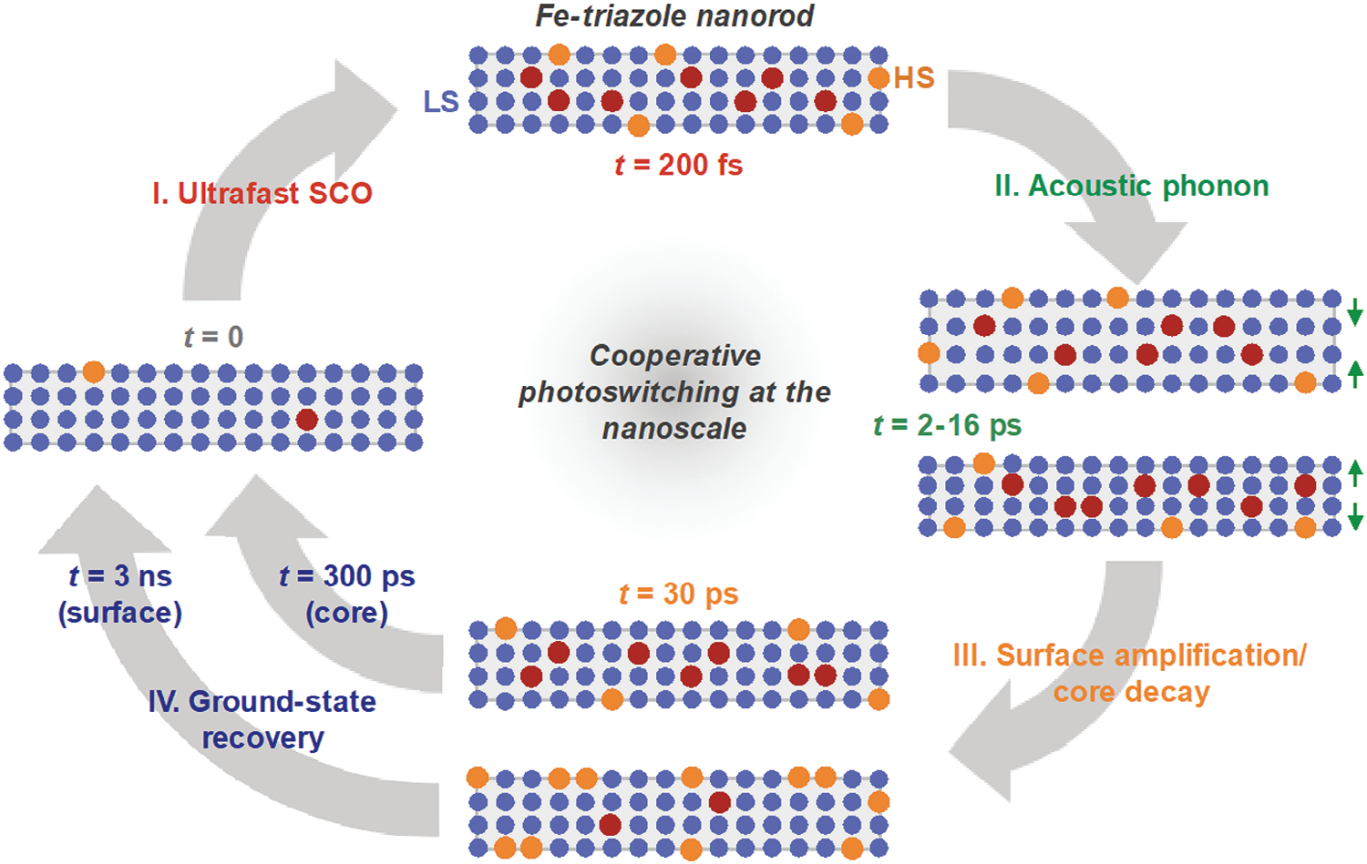
Schematic representation of the SCO dynamics in Fe‐trz nanoparticles.

The important role of surface SCO molecules elaborated here naturally opens the question of how these effects depend on the particle size. The weight of surface molecules on the global properties of the particle is expected to increase with decreasing particle size. **Table** **1** shows that the relative ΔHS contribution of surface species increases as the particle size decreases (see Section S8.4, Supporting Information for details). These findings support an effective shift of the HS population from the nanoparticle core toward the surface on the ≈35 ps time scale (**Figure** **8**). Conversely, the amplification time constants do not show a clear trend as a function of particle size (Figure S45, Supporting Information). The invariance with particle size suggests we cannot equate this surface HS amplification to a purely elastically driven process as the aforementioned “elastic amplification” has been suggested to be.^[^ ^6^ ^]^ One explanation is that the surface HS amplification is not driven elastically, that is, coincident with the lattice expansion, but requires thermal activation due to local energy barriers.^[^ ^26^, ^102^ ^]^ In this so‐called “bottleneck,” LS→HS conversion necessitates both lattice volume expansion (elastic) and thermal activation (thermal). On small length scales—as in our case—the strain wave propagation will be very rapid (≈1–10 ps) and HS amplification may await thermal activation by the heat transfer from photoexcited centers to their neighbors. At the surface, we expect these thermal diffusion times to be relatively insensitive to particle size (Equation S17, Supporting Information), which may give rise to our size‐independent ≈35 ps timescale. This role of heat in stimulating elastic amplification has led to a shift to discussing these phenomena as “thermoelastic” processes.^[^ ^63^, ^102^ ^]^ Within the mechano‐elastic model, the energy barriers can be represented by the HS→LS activation energy *E*_a_ (Equations S13,S14, Supporting Information) and previous simulations^[^ ^26^ ^]^ have shown a delayed HS transformation in agreement with this “bottleneck” hypothesis.

**Table 1:** Size dependence of the relative fractions of core and surface HS species after amplification. The core fraction is determined from the subtraction: *A*_C_ − *x*_C_ *A*_C_. The surface fraction is determined from the addition: *A*_S_ + *x*_S_ *A*_S_. Details are given in Section S8.4 (Supporting Information).

Fe center location	Small (1)	Medium (2)	Medium (2*)	Large (3)
Core	40.8 ± 2%	46.3 ± 2.3%	45.5 ± 1.8%	53 ± 2%
Surface	59.2 ± 0.8%	53.7 ± 1%	54.5 ± 0.8%	47 ± 1%

## 3 Conclusion

Using a global and target fit analysis of the OTA data in the UV probing region, we were able to spectrally and kinetically isolate the HS population dynamics at the surface and in the core of strongly cooperative SCO nanoparticles. Supported by mechano‐elastic MC simulations, we show that the HS molecules at the surface of the nanoparticles decay on a longer time scale than those in the core, due to a lower local pressure and smaller ligand field. Furthermore, upon homogeneous photoexcitation, a transient (≈35 ps) increase in the HS population at the surface of the nanoparticles occurs, while the population in the core of the nanoparticles decays concomitantly. This shift of the HS population moves the lattice toward a lower elastic energy and restoration of mechanical equilibrium. The total HS population decays monotonically, as expected under the low‐excitation conditions of the experiment. We demonstrate that the acoustic nanorod breathing period scales with the size of the nanoparticles, while the HS (de)amplification time scales do not show an obvious size dependence. A clear dependence on nanoparticle size appears in the ΔHS fraction after the amplification event, supporting our spectro‐kinetic fitting model and assignment of surface and core HS dynamics. The identified surface species shows a relatively stronger d–d absorption band, as predicted by DFT calculations for distorted model molecules. A summary of the sequence of steps in the HS dynamics after photoexcitation is schematically shown in Figure 8.

Our results show that the dynamics of molecular switching (LS→HS and HS→LS) differ at the surface and in the core of nanoscale SCO materials due to the difference in local energy barriers. The amplification of the HS population at the surface is rather unexpected, given the low excitation conditions and the fact that the surface is usually associated with a reduction of cooperativity at the nanoscale.^[^ ^20^, ^21^, ^22^, ^23^ ^]^ We think a combination of circumstances gives rise to the observation of this phenomenon in the present study: 1) the unusually strong cooperativity of the Fe‐trz material, even for the smallest nanoparticle sizes; 2) its specific 1D‐polymeric structure with the Fe‐trz chains aligned along the long nanorod axis; 3) the fact that we conduct our photoswitching experiments far below the phase transition, which avoids significant macroscopic thermal SCO effects; 4) the particular sensitivity of metal‐centered d‐d transitions to slight changes in the ligand‐field strength and symmetry; and 5) the impulsive excitation with fs UV pulses, imparting >4 eV of excess energy to the absorbed molecule.

The focus of this study was on nanoscale spin‐transition materials. However, we believe that our results have profound implications for the dynamical size limits of photoswitching in a wide family of phase transition materials displaying strong electron‐lattice coupling.^[^ ^103^ ^]^ The common feature of these materials is the central role of elasticity and a significant volume change upon the phase transition. Future investigations on such nanoscale volume‐changing materials, for example, under varying light excitation conditions, as a function of temperature, for nanoparticles embedded into different matrices, or for particles of different shapes and anisotropy, should deliver further mechanistic handles that can be used for the control and the preservation of cooperativity at the nanometric scale and, ultimately, the rational design of small‐scale functional switching devices.^[^ ^104^ ^]^

## Conflict of Interest

The authors declare no conflict of interest.

## Supporting information



Supporting Information

## Data Availability

The data that support the findings of this study are available from the corresponding author upon reasonable request.
