# Supplementary material for: Size and Surface Effects in the Ultrafast Dynamics of Strongly Cooperative Spin‐Crossover Nanoparticles
Source: Small. 2024 Nov 10;21(2):2405571. doi: 10.1002/smll.202405571 (PMC11735884; doi:10.1002/smll.202405571)
Supplement: Supplementary file 1 — Supporting Information [file SMLL-21-2405571-s001.docx]

Supporting Information

Size and Surface Effects in the Ultrafast Dynamics of Strongly Cooperative Spin-Crossover Nanoparticles

Tyler N. Haddock, Teresa Delgado, Marc Alías-Rodríguez, Coen de Graaf, Cristian Enachescu, and Renske M. van der Veen*

**S1. Synthesis Description**

We synthesized Fe(Htrz)_2_(trz)](BF_4_) (Fe-trz) nanoparticles using the reverse-micelle technique (**Figure S1**), reported previously.^[1–4]^ We adapted the synthesis to achieve size variability, while maintaining a stable dispersion. We discovered that particles could be washed and isolated from the surfactant with ethanol and remain in stable solution after re-dispersion into acetonitrile (MeCN).


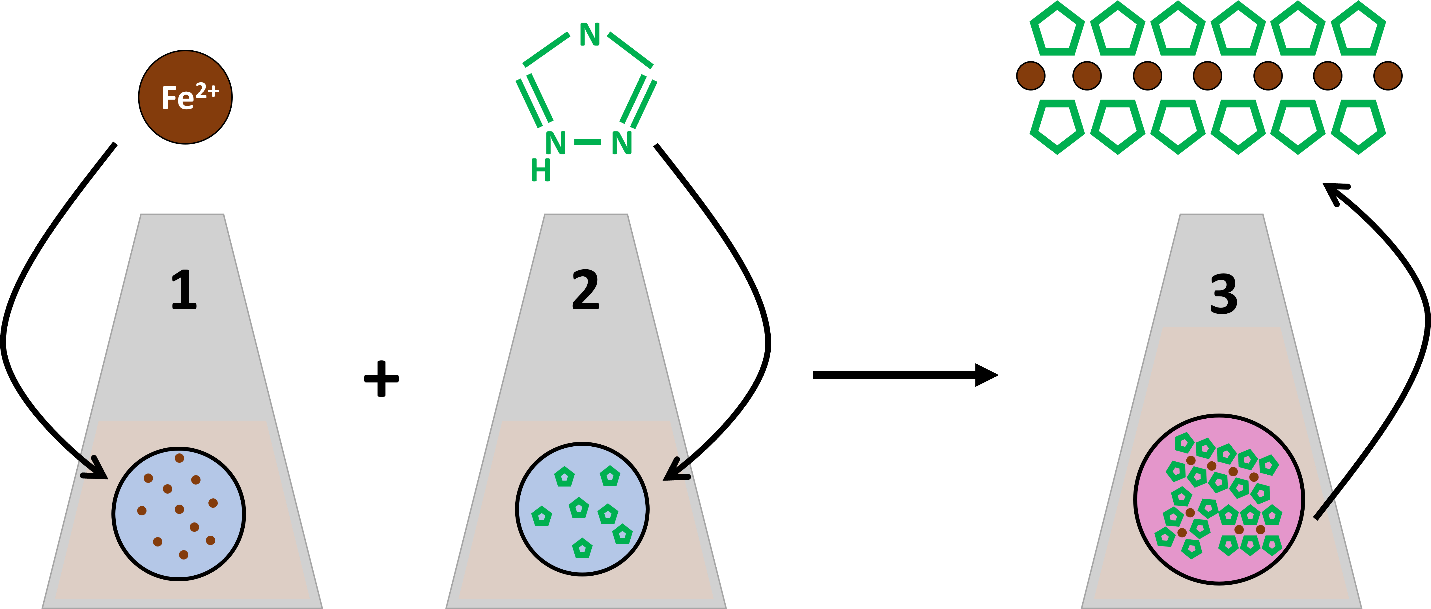


**Figure S1.** Cartoon of the reverse-micelle synthesis of Fe-trz nanoparticles.

**S1.1. Synthetic Steps**

All reactions were performed in air under ambient conditions. The steps below are for batches **2**, **2***, and **2**** (the latter which was used for SQUID only).

1. Two flasks are filled with 50 mL of *n*-heptane each. Flask 1 will contain the iron emulsion and flask 2 will contain the triazole emulsion.
2. 10 g of bis(2-ethylhexyl) sulfosuccinate sodium salt (NaAOT) are stirred to each flask.
3. Several milligrams of ascorbic acid are added to flask 1 to reduce oxidation.
4. The solutions are mixed thoroughly for over 10 minutes to dissolve the surfactant.
5. The aqueous Fe precursor is made by dissolving 1 g of Fe(BF_4_)_2_·6H_2_O into 6 mL of Millipore water. The solution is sonicated for 15 minutes.
6. The aqueous triazole precursor is formed by dissolving 0.6 g of 1H-1,2,4-triazole into 3 mL of Millipore water. The solution is sonicated for 15 minutes.
7. The aqueous Fe solution is pipetted slowly into flask 1 while stirring rapidly.
8. The aqueous triazole solution is pipetted slowly into flask 2 while stirring rapidly.
9. After 5 minutes of mixing, the two emulsions are then combined (we added the Fe solution into triazole solution) while stirring vigorously.
10. The nanoparticle dispersion starts to turn from transparent to clear pink. The final solution is stirred for 10 minutes.

The synthesis of the smaller particles—batch **1**—was the same as above except that 1.5 g of Fe precursor and 0.9 g of triazole were employed. For the large (**3**) particles, the same procedure as above was used except there was a 2-fold reduction of all materials— *n*-heptane, NaAOT, water—in the medium synthesis, but with 0.3125 g of Fe precursor and 0.1875 g of triazole. An additional batch of medium particles was synthesized, but instead of *n*-heptane, *n*-octane was used (due to its high boiling point). This batch was not washed, and temperature-dependent UV-visible absorption was measured to measure the HS absorption spectrum in Figure 1c.

**S1.2. Washing Procedure**

The final nanoparticle/micelle solution was typically stable in *n*-heptane for several days under refrigeration. We developed the following washing procedure to isolate the Nanoparticles from the surfactant:

1. The nanoparticle/micelle solution is dried down, forming a sticky solid.
2. The dried solid is re-suspended in ethanol, producing cloudy solution.
3. The ethanol suspension is centrifuged until the supernatant is clear.
4. The supernatant is discarded and the solid pellet is re-dispersed in fresh ethanol.
5. An additional cycle of centrifuging is performed.
6. If the supernatant is still colorless, steps 4-5 are repeated.
7. After 2 or more centrifugations, the supernatant will remain slightly pink, as some particles remain in solution. At this stage, the supernatant is discarded and the remaining pellet is dispersed in MeCN to be used for measurements.
8. The final solution is stored in a refrigerator to improve the stability.

**S2. Nanoparticle Characterization**

**S2.1. TEM Size Distribution Analysis**

Transmission electron microscopy (TEM) images of the small (**1**), medium (**2** and **2***), and large (**3**) samples are shown in **Figure S2**. The small and medium samples were measured on a Jeol 2100 Cryo. The large sample was measured on a Hitachi 9500.


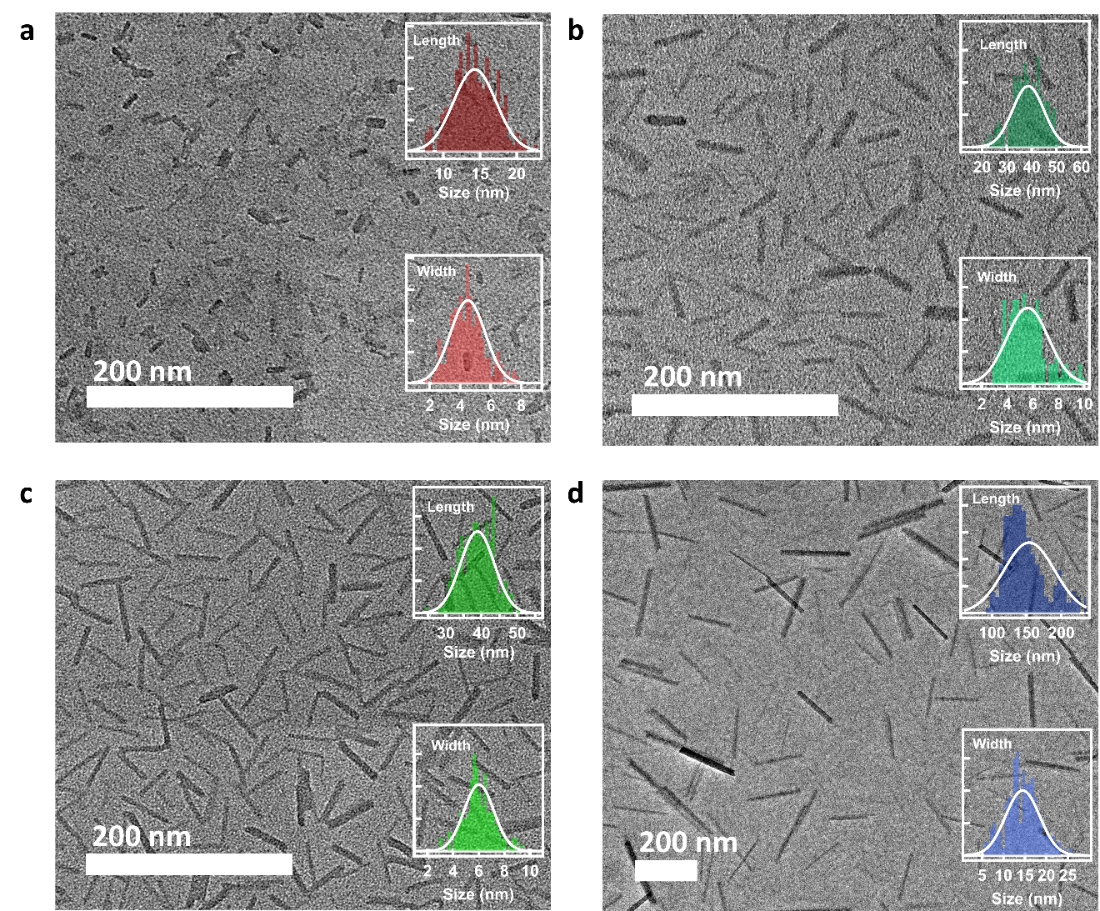


**Figure S2.** TEM images of Fe-trz nanoparticles. a) Small, batch **1**. b) Medium, batch **2**. (c) Medium, batch **2***. (d) Large, batch **3**.

The nanoparticle lengths and widths were measured manually using ImageJ. Between 100 and 300 particles were analyzed in various images sample a good distribution. Size histograms for length and width of the small, medium, and large particles are overlaid in Figure S2 (along with Gaussian fits). **Table S1** shows the particle dimensions analyzed from TEM/ImageJ.

The measured dimensions enable estimation of the predicted number of core and surface Fe unit cells. In the crystal structure of Fe-trz, every 1D chain has 6 neighboring chains in a hexagonal arrangement with two Fe-Fe interchain distances.^[5]^ Using this crystal structure, we approximated the Nanoparticles to be hexagonal prisms. The hexagonal prisms were constructed in CrystalMaker to model the TEM-measured nanorod morphology (**Figure S3**).


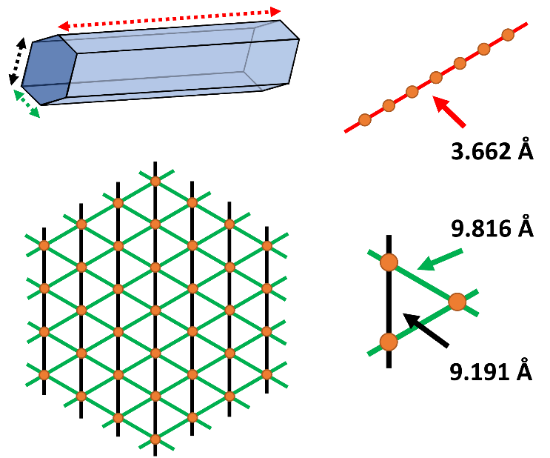


**Figure S3.** Fe-trz nanoparticle model from reported crystal structure.^[5]^

With the CrystalMaker nanorods, we counted the number of unit cells on the surface (compared to total) to give us the mean surface fraction. To estimate the error, we created structures of ± 1 standard deviation about the average width and length from the size analysis. In other words, one structure would be made from 1 standard deviation above the mean length *and* 1 standard deviation above the mean width; another structure would be 1 standard deviation below the mean length and width. In this way, we could give a range of possible surface fractions (shown in Table S1) which took into consideration the size dispersion.

**Table S1.** Distribution of nanoparticles from image analysis. Estimated number of unit cells and surface percent is also shown.

|  | **Length (nm)** | **Width (nm)** | **No. Fe Atoms** | **Estimated Surface Percent** |
| --- | --- | --- | --- | --- |
| Small (**1**) | 14.3 ± 2.9 | 4.5 ± 1.1 | 1200 | 55.2-66.8% |
| Medium (**2**) | 38.6 ± 6.3 | 5.6 ± 1.6 | 5088 | 37.1-58.9% |
| Medium (**2***) | 38.9 ± 4.7 | 6.0 ± 1.2 | 5616 | 37.0-49.3% |
| Large (**3**) | 153.8 ± 36.6 | 14.5 ± 4.0 | 58237 | 17.9-26.7% |

**S2.2. SQUID**

The phase transition temperature and hysteresis of Fe-trz batches were characterized with a Quantum Design MPMS3 superconducting quantum interference device (SQUID) magnetometer. SQUID was performed to measure temperature (*T*) dependence of the magnetic moment, **m** in for multiple cycles about the hysteretic LS↔HS phase change in Fe-trz. For each measurement, $\sim$10 mg of dried and crushed Fe-trz powder (from the washed MeCN solution) was used. The temperature ramp rate was set at 0.5 K/min. Based on a rate dependence study using dynamic thermal analysis, this rate is sufficient to avoid large kinetic effects in the phase transition temperatures. The total molar magnetic susceptibility, $\chi_{mol}^{\mathrm{Tot}}$ is calculated using

$\chi_{\mathrm{mol}}^{\mathrm{Tot}}=\frac{M}{m}\frac{\mathbf{m}}{\mathbf{H}}$ (S1)

where *m* is the mass of the sample measured, **H** is the magnetic field strength (2000 Oe for every measurement), and *M* is the molar mass of an Fe unit cell. The paramagnetic part of the total magnetic susceptibility, *χ*_mol_ is calculated correcting for the negative diamagnetic contribution using Pascal’s constants.^[6]^ **Figure S4** shows *χ*_mol_*T* as a function of *T* for 3 consecutive of cycles of the small batch (**1**). Irreversible changes occur during the first heating cycle, which is why it deviates from further ramp cycles.^[7]^


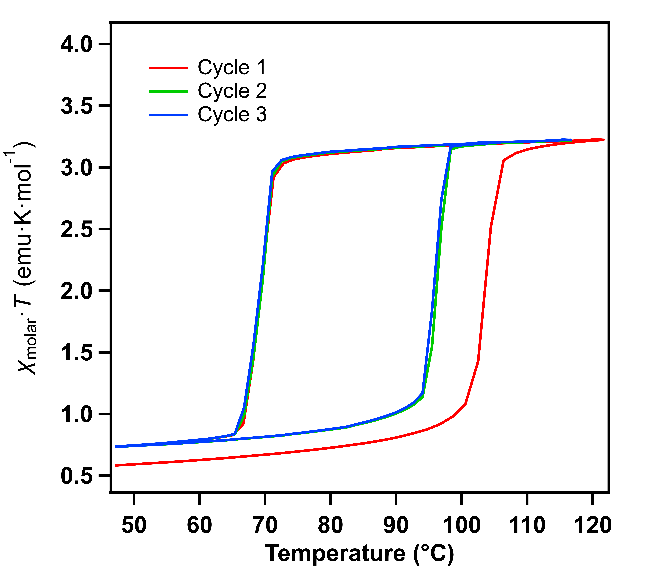


**Figure S4.** The temperature dependence of *χ*_mol_*T* for batch **1**.

**Figure S5** shows the *χ*_mol_*T* as a function of *T* for batches of small (**1**), medium (**2****), and large (**3**) particles during the third thermal cycles. For the large particles, the *χ*_mol_*T* value in the HS phase of 4 emu·K·mol^-1^ is far from the typical range of 3-3.5 emu·K·mol^-1^. We suspect this is due to a systematic measurement error of sample mass (see Equation S1).


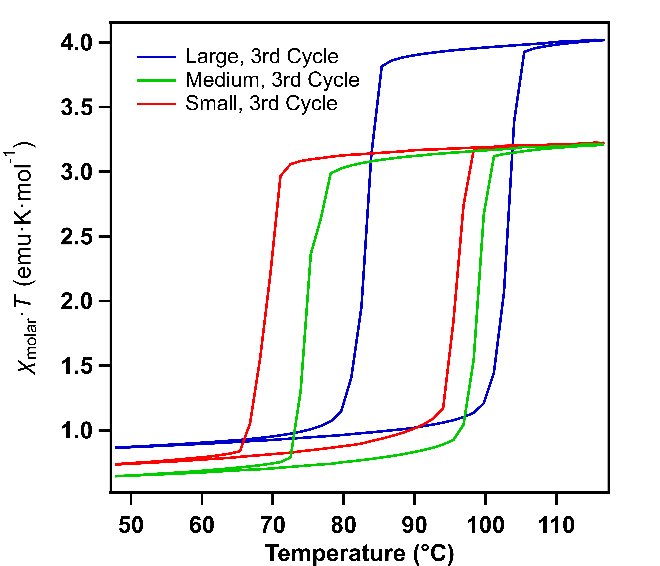


**Figure S5.** The temperature dependence of *χ*_mol_*T* for third cycle of small, medium, and large batches.

In **Table S2**, the heating and cooling transition temperatures and hysteresis widths (Δ*T*) for several batches of small, medium, and large particles are shown. SQUID measurements of batches **2** and **2*** were not high quality and thus not shown in Figure S5, but are included in Table S2.

**Table S2.** The heating and cooling phase transition temperatures as well as the hysteresis width.

|  | $\boldsymbol{T}_{\mathbf{1}\mathbf{/}\mathbf{2}}^{\boldsymbol{\uparrow}}$ **(K)** | $\boldsymbol{T}_{\mathbf{1}\mathbf{/}\mathbf{2}}^{\boldsymbol{\downarrow}}$ **(K)** | **Δ*T* (K)** |
| --- | --- | --- | --- |
| Small (**1**) | 369.3 | 342.0 | 27.3 |
| Medium (**2**) | 368.9 | 345.9 | 23.1 |
| Medium (**2***) | 370.1 | 344.9 | 25.1 |
| Medium (**2****) | 372.2 | 347.8 | 24.4 |
| Large (**3**) | 375.8 | 356.1 | 19.7 |

**S2.3. UV-visible Spectroscopy**

UV-visible absorption was measured with a Varian Cary 5G spectrophotometer at 1 nm intervals. The UV-visible absorption spectra of the 4 batches of washed nanoparticles (MeCN, RT) are shown in **Figure S6-S9**.


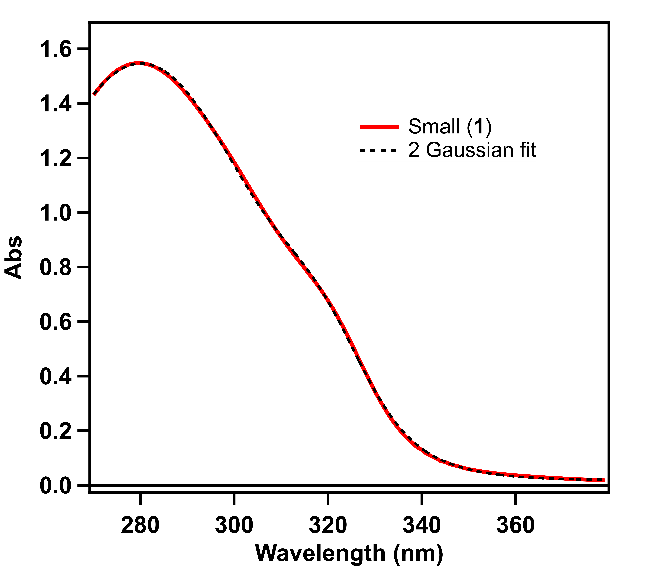


**Figure S6.** UV-Vis spectra of batch **1** with 2 Gaussian fit.


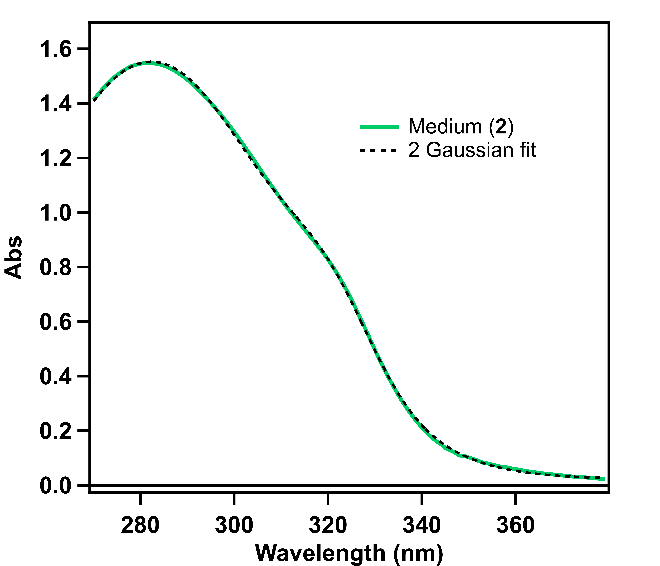


**Figure S7.** UV-visible spectra of batch **2** with 2 Gaussian fit.


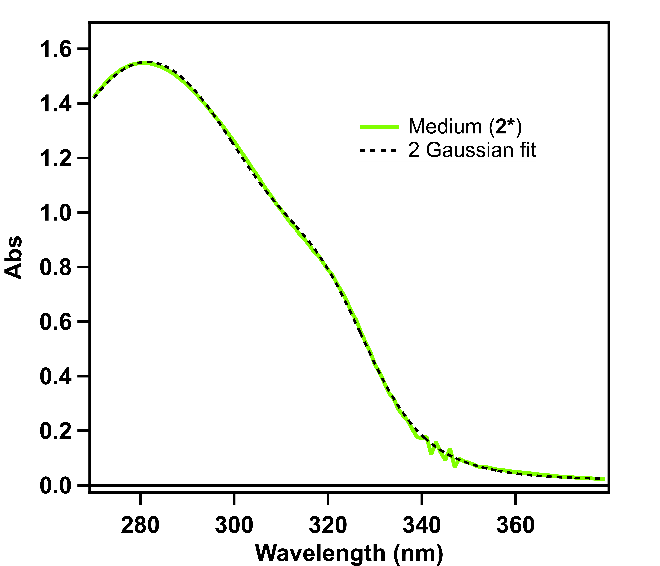


**Figure S8.** UV-visible spectra of batch **2*** with 2 Gaussian fit.


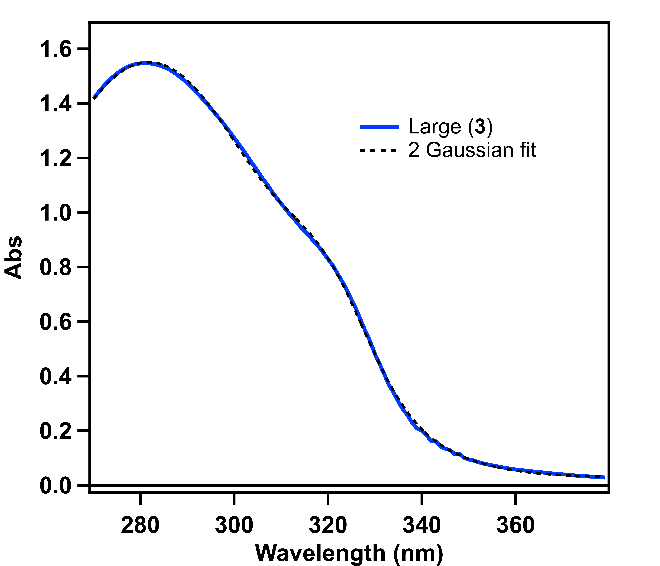


**Figure S9.** UV-visible spectra of batch **3** with 2 Gaussian fit.

In order to quantify spectral changes, we fit our UV-visible (and later transient spectra) with a 2-peak function to account for the two relevant electronic transitions: ^1^A_1g_→^1^MLCT and ^1^A_1g_→^1^T_2g_ bands. We chose a 2 Gaussian fit in wavelength-space as it performed better than other functions:

$f\left( \lambda\right)=A_{1}\frac{1}{\sigma_{1}\sqrt{2\pi}}e^{-\frac{\left( \lambda-\mu_{1} \right)^{2}}{2{\sigma_{1}}^{2}}}+A_{2}\frac{1}{\sigma_{2}\sqrt{2\pi}}e^{-\frac{{(\lambda-\mu_{2})}^{2}}{2{\sigma_{2}}^{2}}}$ (S2)

where *A*, *μ*, and *σ* are the area, center wavelength, and standard deviation of each Gaussian component as a function of wavelength, *λ*. The subscripts 1 and 2 designate the ^1^A_1g_→^1^MLCT and ^1^A_1g_→^1^T_2g_ bands, respectively. These fits are shown in the aforementioned UV-visible spectra and the results are included in **Table S3**.

**Table S3.** The results of the fit using Equation S2 on the UV-visible absorption of batches **1**, **2**, **2***, and **3**.

|  | ***μ*_1_ (nm)** | ***σ*_1_ (nm)** | ***A*_2_/*A*_1_** | ***μ*_2_ (nm)** | ***σ*_2_ (nm)** |
| --- | --- | --- | --- | --- | --- |
| Small (**1**) | 280.16 ± 0.07 | 25.7 ± 0.2 | 0.044 ± 0.002 | 319.2 ± 0.1 | 8.9 ± 0.2 |
| Medium (**2**) | 282.3 ± 0.08 | 27.6 ± 0.2 | 0.045 ± 0.002 | 321.0 ± 0.1 | 9.3 ± 0.2 |
| Medium (**2***) | 281.5 ± 0.1 | 26.7 ± 0.3 | 0.052 ± 0.004 | 320.2 ± 0.2 | 9.2 ± 0.3 |
| Large (**3**) | 281.8 ± 0.1 | 27.2 ± 0.2 | 0.050 ± 0.003 | - 1. ± 0.1 | 8.8 ± 0.2 |

**S3. Broadband OTA Spectroscopy**

**S3.1. Description of the Experiment**


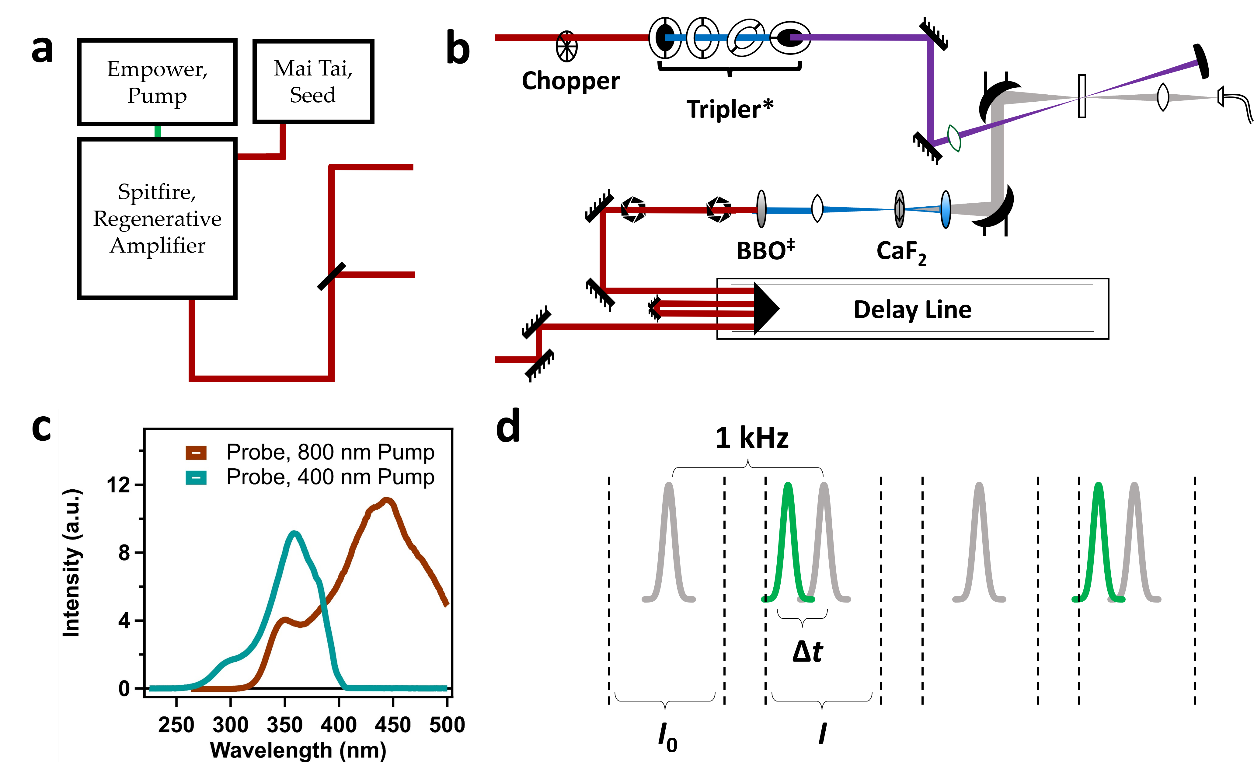


**Figure S10.** a)-b) Femtosecond OTA setup. c) Broadband probe spectra. d) Pump-probe pulse sequence.

A femtosecond pump-probe setup was used to perform optical transient absorption (OTA) measurements. **Figure S10** shows a diagram of the setup. The pump and probe beams are split from the same 800 nm beam generated by a Ti:Sapphire regenerative amplifier (1 kHz, 130 fs, Spitfire, Spectra-Physics). The smaller fraction generates the probe. A broadband UV probe was generated by doubling the pulse to 400 nm and focusing in a translating CaF_2_ crystal, generating a supercontinuum.^[8]^ Pumping the CaF_2_ with 400 nm (instead of 800 nm) shifts the supercontinuum to the UV (see Figure S10b), and enabled investigation of the ^1^A_1g_→^1^MLCT transition. The pump beam is chopped to 500 Hz. and tuned to 267 nm using a tripler.

At the sample position, the probe beam was typically ~40 μm 1/e^2^ diameter and the pump was then tuned to at least 3 times larger. The transmitted beam was focused into a multimode fiber and coupled into the CMOS detector (Ultrafast Systems). A custom built LabVIEW-based data acquisition and diagnostics program was used to setup measurements and determine the transient absorption signal, ∆*A*(λ, t).

All experiments were performed in transmission geometry with the samples flowed through a quartz flow cell to refresh faster than 1 kHz. OTA was performed on colloidal solutions of washed Fe-trz Nanoparticles in MeCN. The nanoparticles were measured as is after the washing procedure. While flowing, the sample reservoir was cooled with an ice bath, giving a temperature at the sample position of approximately 288 K. The particles were excited at 267 nm and probed with a broadband white light beam spanning 250 to 390 nm.

The time-resolution for the measurement was estimated by fitting the MeCN cross phase modulation (CPM) response to the following function:

$f\left( t \right)=(A_{1}+A_{2}t+A_{3}t^{2})e^{-\frac{\left( t-t_{0} \right)^{2}}{F/2\sqrt{\ln2}}}$ (S3)

where *F* is the full-width at half maximum (FWHM) of the instrument response function (IRF). The amplitudes *A*_1_, *A*_2_, and *A*_3_ are polynomial coefficients which scale the oscillations in the CPM signal around time zero, *t*_0_. The results of this fit are shown in **Figure S11**a, with the values of *F* as a function of the probe wavelength shown in Figure S11b.


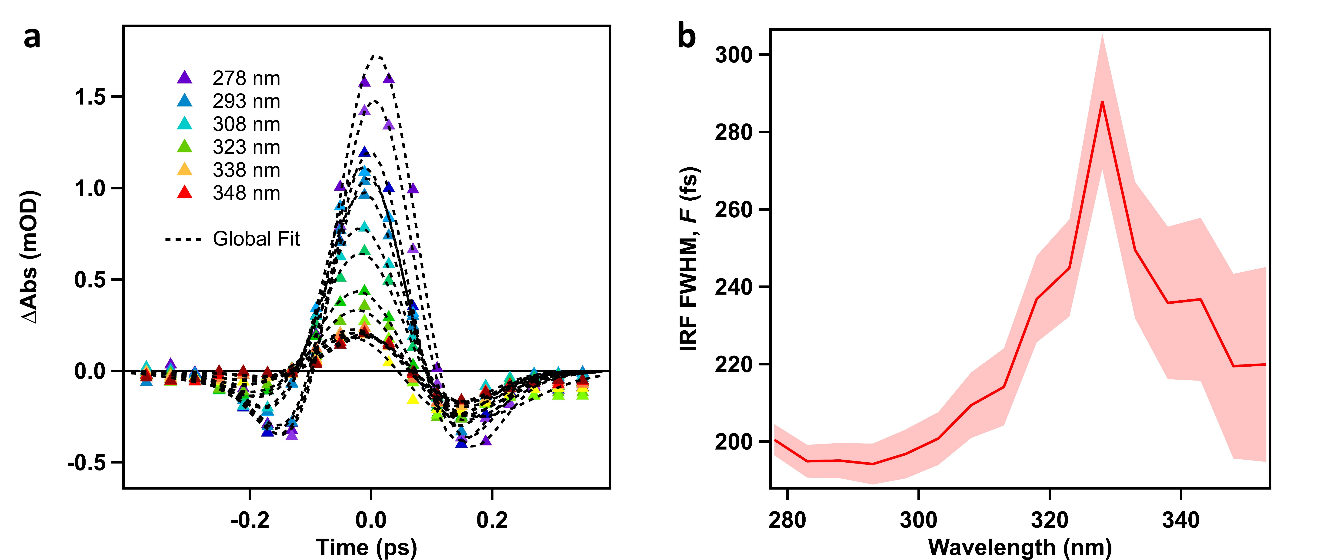


**Figure S11.** a) Kinetic traces at various wavelengths for a MeCN OTA measurement. The CPM fit is shown in dashed lines. b) The probe wavelength dependence of the IRF, *F*. The shaded bounds represent 1 standard deviation.

**S3.2. Correction of Broadband OTA Data for Solvent ESA Absorption**

OTA on spin-coated Fe-trz films showed only negative bleaches of the ground state transitions in the UV. In MeCN solution measurements, we observe a weak, spectrally flat excited state absorption (ESA) across the UV probe region. **Figure S12**a shows scans of MeCN solution, revealing that this effect is due to the solvent. We did not observe the ESA in empty cells.

To mitigate the effect the ESA had on the analysis of the Fe-trz signals, we have subtracted kinetic traces at 360 nm (where only the MeCN ESA is present) from the rest of the transient plot. Figure S12b shows how this subtraction affects transient spectra at 4 ps. All data had this small ESA component subtracted before analysis.


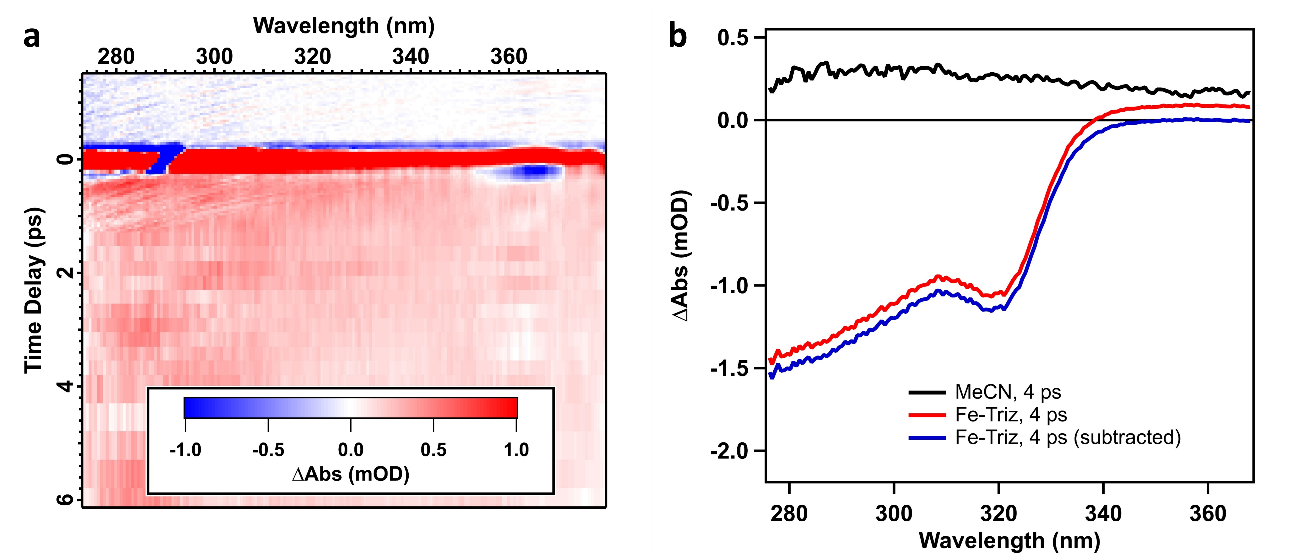


**Figure S12.** a) Transient absorption plot for MeCN excited at 267 nm. b) Transients at 4 ps before and after subtraction of 360 nm kinetic trace.

**S3.3. Results for All Sizes**

The ∆*A*(*λ*, *t*) OTA signal was time-corrected for group velocity dispersion and subtracted for the pre-time zero background and the 360 nm ESA signal discussed above. Before modelling, the CPM artifact was removed to avoid its influence on the fit. The 2D wavelength:time OTA for samples **1**, **2**, **2***, and **3** are shown in **Figure S13-S16**.


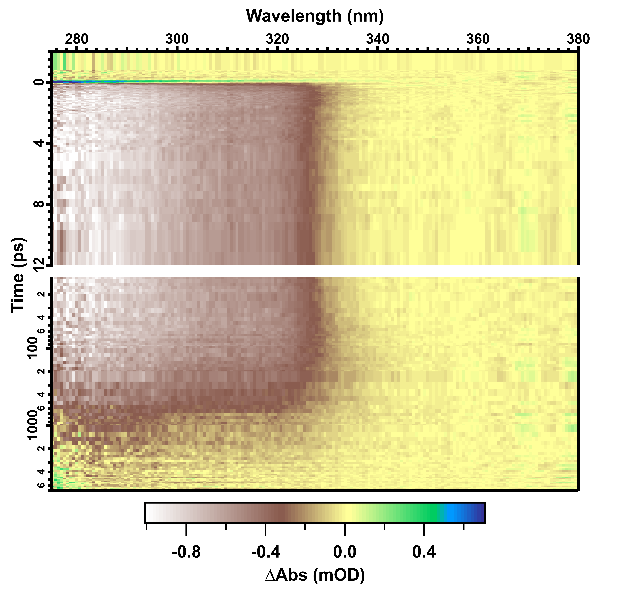


**Figure S13.** OTA for sample **1**.


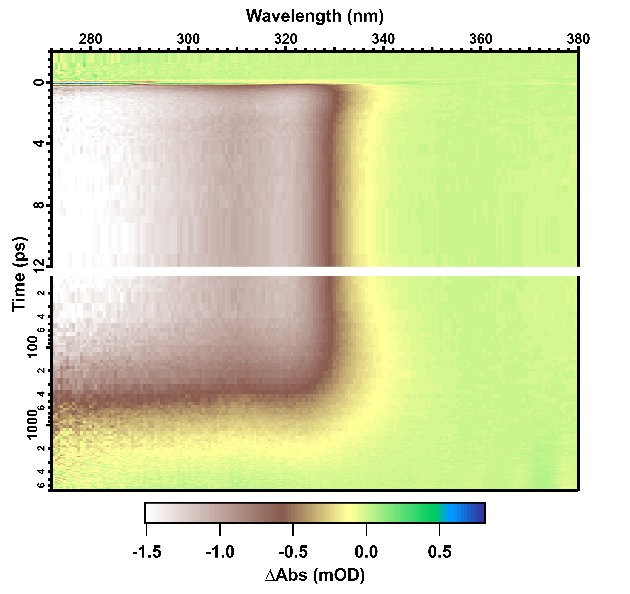


**Figure S14.** OTA for sample **2**.


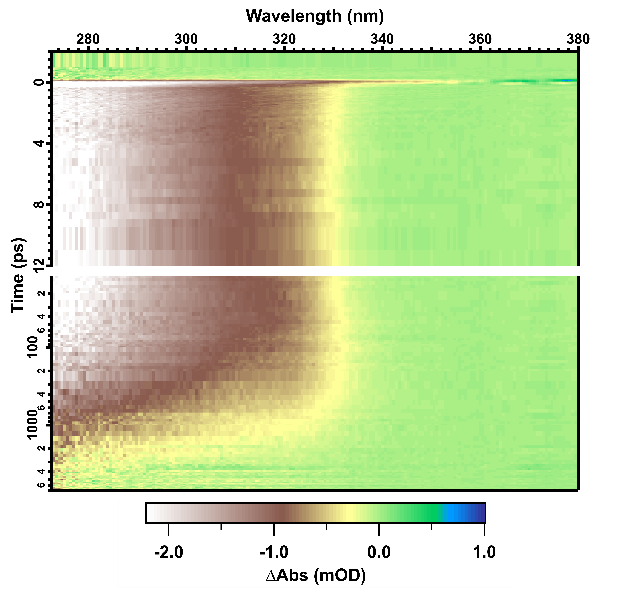


**Figure S15.** OTA for sample **2***.


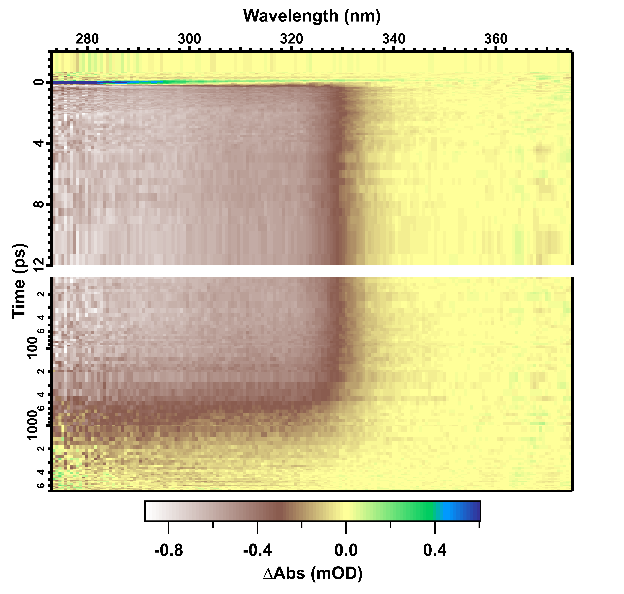


**Figure S16.** OTA for sample **3**.

**S3.4. Estimation of Excitation Yield**

The peak magnitude of the ground state bleach of the ^1^A_1g_→^1^MLCT band was used to estimate photoinduced HS population, $\Delta X_{\mathrm{HS}}^{h\nu}$. We chose this spectral region because the HS species’ charge-transfer band (^5^T_2g_→^5^MLCT) is relatively weak compared with the LS state (Figure 1c). Furthermore, the ^1^MLCT band is less affected by surface/core effects (see DFT section below).

Our OTA detection scheme allowed in situ UV-visible absorption to be measured because the raw intensity of our probe, *I* is measured for every shot. Before and after each measurement, we also measured a MeCN solvent reference intensity, *I*_0_. The ground state absorbance, *A* was then calculated using log_10_(*I*_0_/*I*). Kinetic traces were produced from averaging traces at 281 ± 2.5 nm (small and medium) and 282 ± 2.5 nm (large). The HS fraction was then calculated by referencing transient absorption signal (at around 5 ps delay to avoid the acoustic oscillation) to the UV-visible data as discussed. The excited HS fractions for the 4 batches are shown in **Table S4**.

**Table S4.** The photo-induced HS excitation fraction for batches **1**, **2**, **2***, and **3**.

|  | $\boldsymbol{\Delta}\boldsymbol{X}_{\mathbf{HS}}^{\boldsymbol{h\nu}}$ **(%)** |
| --- | --- |
| Small (**1**) | 0.65 ± 0.02 |
| Medium (**2**) | 0.86 ± 0.03 |
| Medium (**2***) | 0.46 ± 0.02 |
| Large (**3**) | 1.10 ± 0.04 |

These low excitation fractions keep us well within the low excitation regime (<2%), where we observe negligible elastic amplification effects that increase the total HS fraction.

**S4. Femtosecond OTA and Extreme Ultraviolet (XUV) Spectroscopy on Nanoparticle Films**

Ground state and transient extreme ultraviolet (XUV) spectroscopy was performed in collaboration with Professor Josh Vura-Weis and Dr. Ryan Ash. The measurements implemented the Vura-Weis group’s high harmonic generation (HHG) table top instrument which produces femtosecond XUV pulses.^[9]^ The Vura-Weis instrument is capable of measuring the Fe M-edge region, making it an excellent technique for disentangling the ^1^A_1g_, ^3^T, and ^5^T_2g_ excited states of Fe(II) compounds.^[10]^

We prepared thin films by spin coating washed Fe-trz solution onto previously made thin films of polyvinyl chloride (PVC).^[11]^ The UV-visible absorption spectrum of these loaded and unloaded films is shown in **Figure S17**.


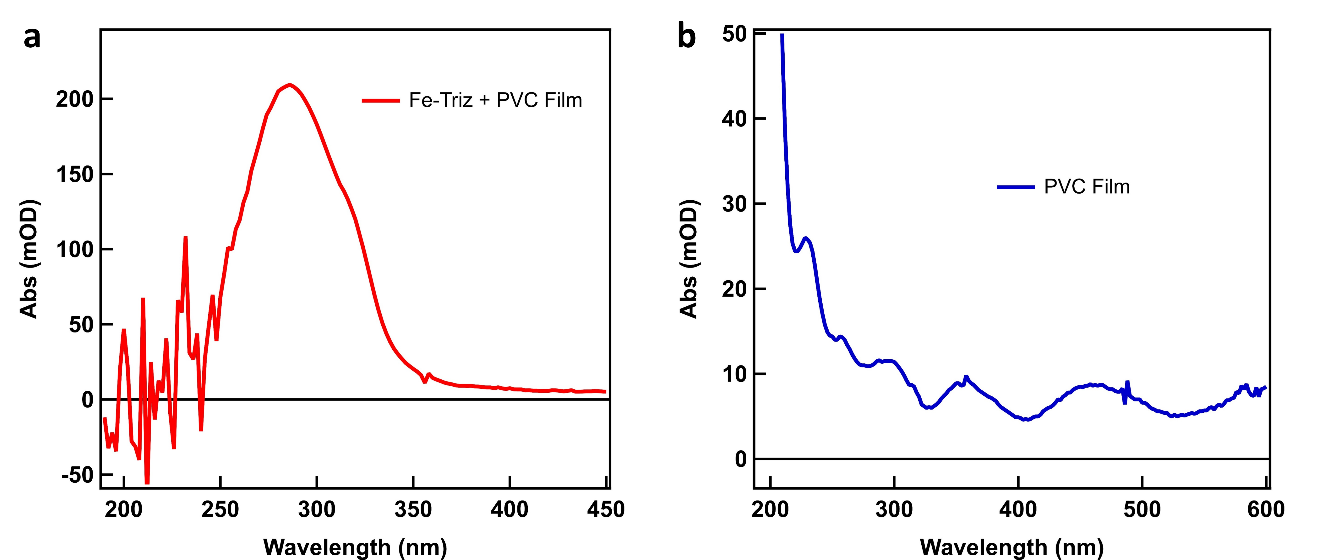


**Figure S17.** a) UV-visible absorption of Fe-trz PVC films. b) UV-visible of unloaded PVC film. Oscillations show thin film interference.

The ground state Fe-trz M-edge absorption is shown in **Figure S18**, where the ligand absorbance was subtracted out. The three peaks (58.7, 61.5, and 68.0 eV) are due to different ^1^A_1g_→^1^A_1u_ excitations^[10]^ and are characteristic of LS Fe(II) compounds.^[12]^


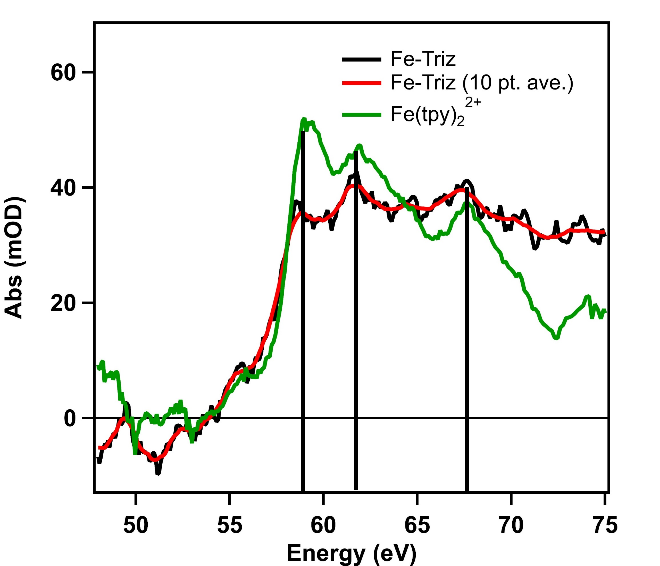


**Figure S18.** Ground state M-edge absorption spectrum of Fe-trz (with and without smoothing) and Fe(tpy)_2_^2+^.

Prior to time-resolved XUV, we carried out OTA on Fe-trz thin films to ensure they behaved the same as solution phase measurements. A representative 2D OTA image plot is shown in **Figure S19**a. Transient spectra at various time delays is shown in Figure S19b. We see clear bleaches of the ground state ^1^A_1g_→^1^MLCT and ^1^A_1g_→^1^T_2g_ transitions. The ground state recovers on the nanosecond timescale, which is consistent with out solution phase measurements.


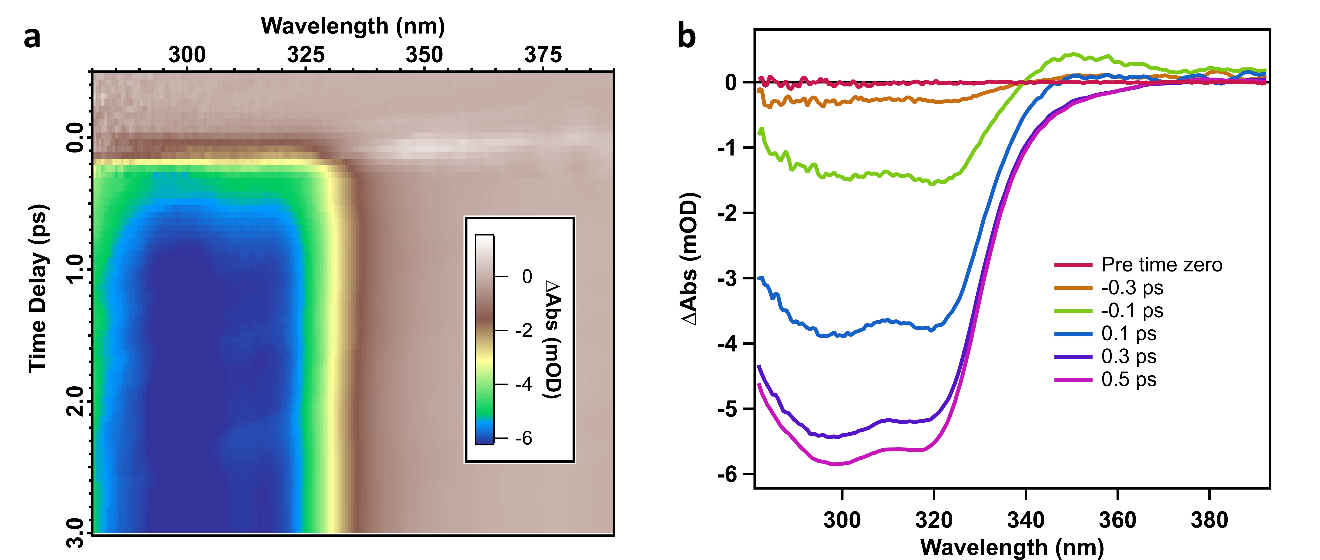


**Figure S19.** a) Transient absorption map of Fe-trz thin films. b) Transient spectra showing ultrafast excited state formation.

The IRF of the thin film OTA measurement was determined to be 170 ± 10 fs (FWHM) by fitting a Gaussian function a kinetic trace at 380 nm (**Figure S20**a). From this IRF, we fit kinetic traces at 300 nm to an erfexpRise function (Equation S5). This gave us an excited state formation time of 210 ± 10 fs, just over the IRF (Figure S20b).


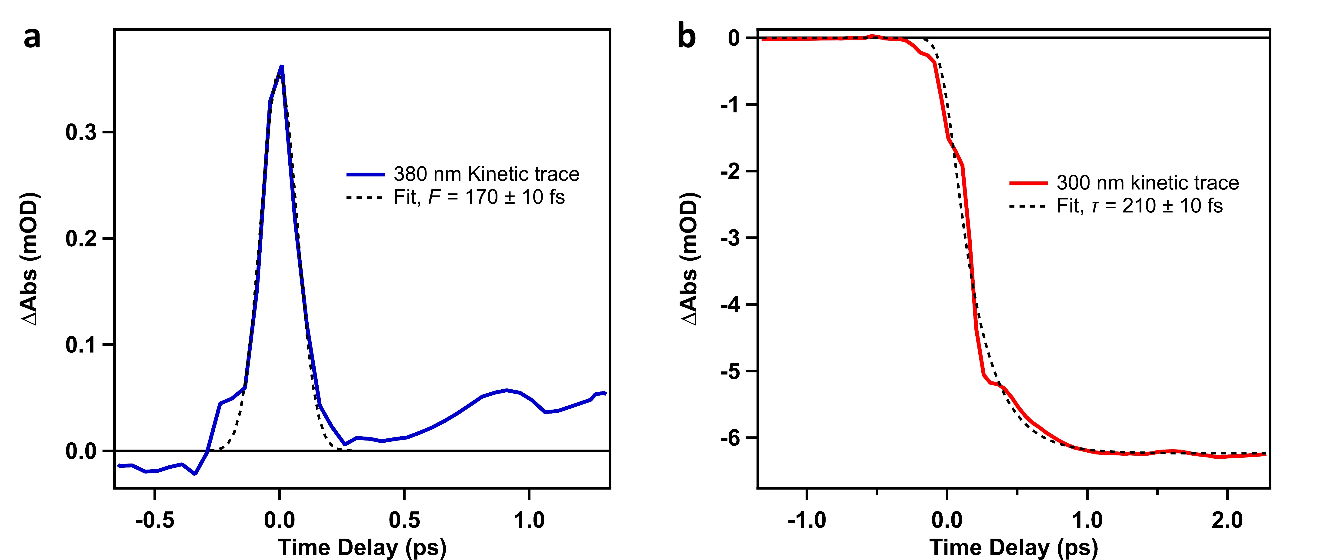


**Figure S20.** a) OTA kinetic trace of Fe-trz film at 380 nm. b) OTA kinetic trace of Fe-trz film at 300 nm.

To confirm the identity of the OTA excited state as the HS state and determine if any intermediate states can be observed, we performed transient M-edge spectroscopy on the Fe-trz films. A tripler as added to excite the ^1^MLCT band at 267 nm and we measured from -0.5 to 50 ps. Transient XUV absorption spectra at different time delays are shown in **Figure S21**a. It is clear from the plot that after excitation into the ^1^MLCT, Fe-trz decays into the ^5^T_2g_ (HS) state within 100’s of femtoseconds. Figure S21b shows the spectra at 1 ps compared to the reported quintet - singlet difference spectra of two Fe(II) molecules (Fe(phen)_3_ and Fe(bpca)_2_) with good agreement.^[10,12]^ The ^5^T_2g_ – ^1^A_1g_ difference spectra in Fe(II) molecules have a characteristic peak at 57 eV due to a red-shift and 3-fold increased peak strength in the HS state compared to the LS.


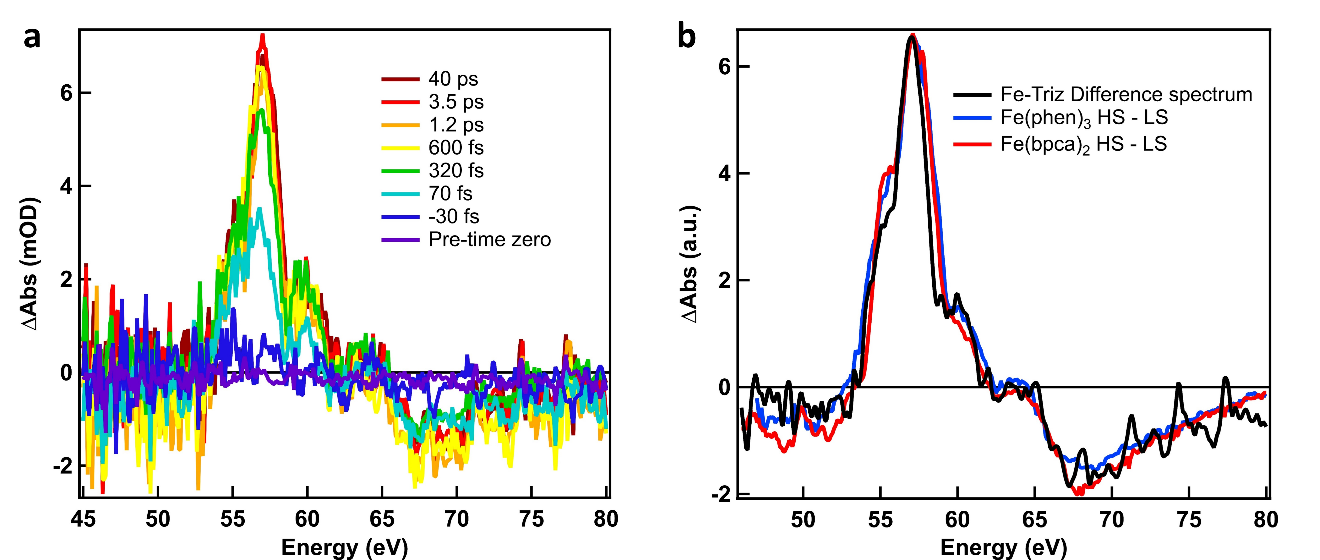


**Figure S21.** a) Transient XUV absorption spectra at different time delays. b) Comparison between Fe-trz final excited state species with quintet difference spectra of two Fe(II) molecules.^[10,12]^

The spectral slices do not show clear evidence of a ^3^T intermediate state. One hallmark of the ^3^T transient is the presence of an ESA between 63 and 65 eV, where the HS transient is near zero.^[10]^ In **Figure S22**, we plot this energy region along with regions characteristic of the quintet state (57 eV) and MLCT/quintet states (54.5 eV). The 63-65 eV regions shows no observable ESA above the noise. The quintet and MLCT/quintet region kinetic traces were fit to an exponential rise convoluted with the IRF (170 fs FWHM determined from hematite measurements). The results of the fit show an earlier (∆*t*_0_ = 60 ± 60 fs) and faster (∆*τ* = 40 ± 70 fs) rise for the MLCT/quintet kinetic trace compared with the quintet trace. However, the parameters are within 1 standard deviation of each other and are thus unconclusive. The absence of a ^3^T from observation could be due to the 170 fs IRF of the measurement, which is four times longer than the ^3^T state lifetime for (Fe(phen)_3_).^[10]^


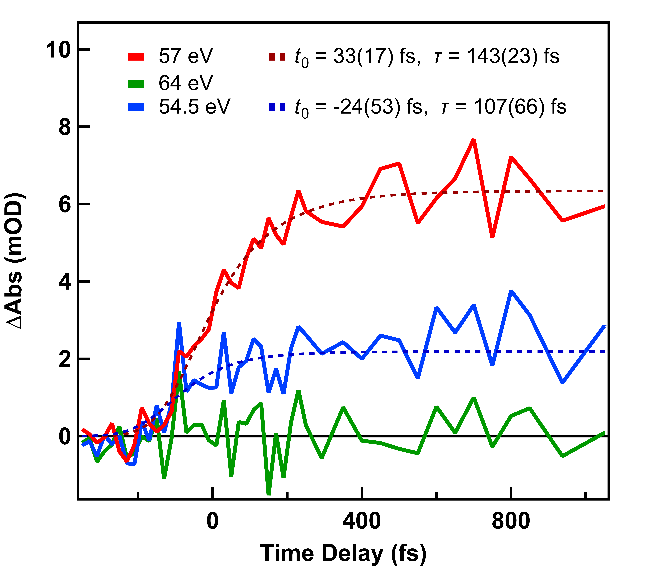


**Figure S22.** Time-resolved XUV kinetic traces at 54.5, 57, and 64 eV. The dashed lines show the results of an erfexpRise fit.

The femtosecond XUV and OTA experiments on thin films provide a clear case that following ^1^MLCT excitation, Fe-trz nanoparticles undergo ultrafast (<200 fs) relaxation into the ^5^T_2g_ HS state (**Figure S23**).


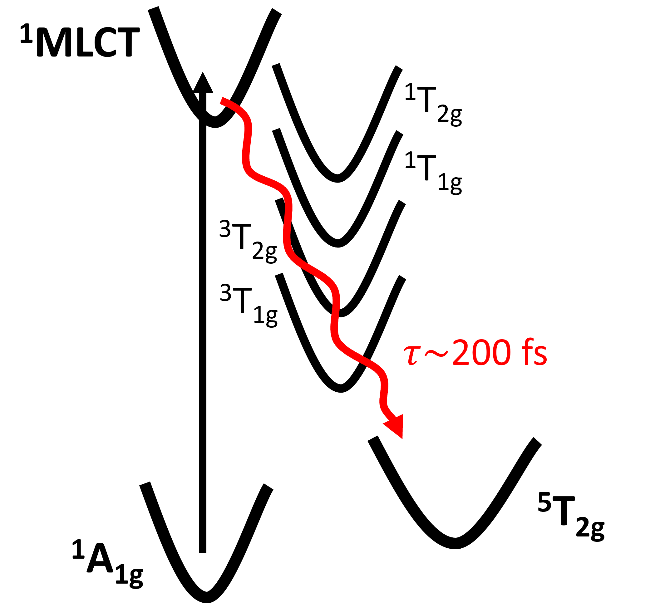


**Figure S23.** Fe-trz ultrafast photophysics after ^1^MLCT excitation.

The success of these measurements—utilizing the PVC polymer film preparation method^[11]^—are promising for the measurement of other spin-cast nanoparticle systems using table-top XUV spectroscopy.

**S5. Analysis of the Acoustic Oscillation by Global Fitting**

**S5.1. Linear Dependence of Oscillation Period with Laser Fluence**

At the sub 1% excitation fractions in our OTA, we have insufficient signal to noise ratio to analyze the acoustic oscillations in our data. In order to verify that using higher excitation data simply scales the oscillation amplitude, we have analyzed batch 2 at 4 excitation fractions. Using 305 nm kinetic traces at 0.5%, 4%, 9%, and 12% excitation, we performed a global fit using the Equation S4 in the following section (**Figure S24**). Amplitude parameters were local, and all others were globally fit.


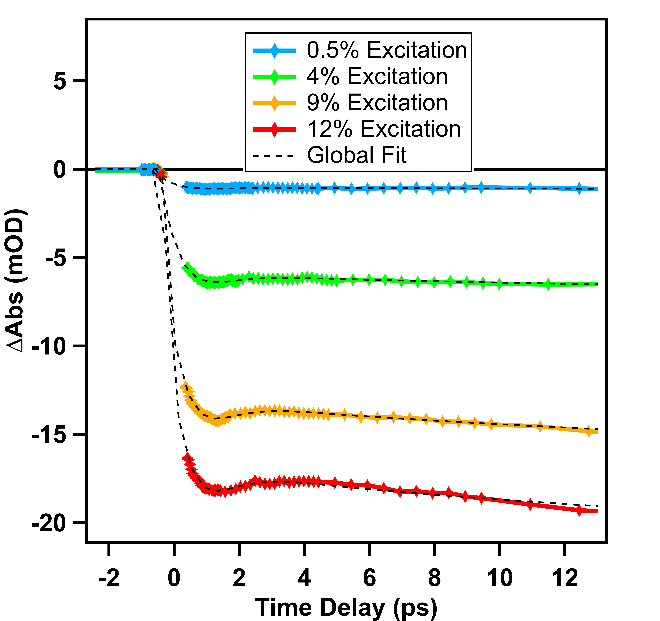


**Figure S24.** Global fit of the 305 nm kinetic traces of batch **2** for 4 excitation fractions.

From this global fit, we have plotted in **Figure S25** the magnitude (absolute value of the amplitude) of the oscillation versus the magnitude of the excitation step jump. I.e., we plotted *A*_1_ versus *A*_2_. We have fit this this to a line, showing both excellent linearity and that the *y*-intercept is very close to 0. Therefore, we have introduced higher power scans to improve the statistics of the analysis of the acoustic oscillation.


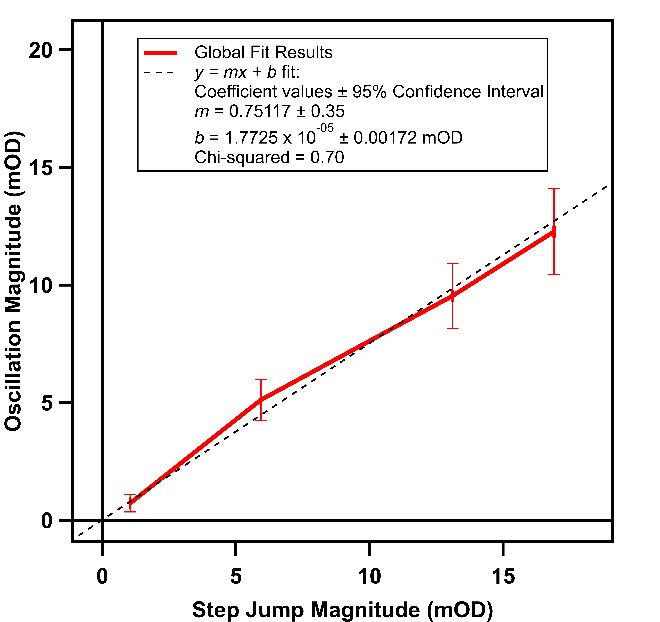


**Figure S25.** Comparing how the oscillation amplitude scales with excitation magnitude.

**S5.2. Details of the Fitting Procedure**

The acoustic oscillation was analyzed using kinetic traces at 305 nm (for each size) which were fit using the following equation:

$$\Delta A\left( t \right)=A_{1}\left[ e^{-\frac{t-t_{0}}{\tau_{\mathrm{damp}}}}\cos\left( {2\pi t}/T+\phi\right)\mathrm{erfexp}\left( F,t_{0},\tau_{\infty}=\infty,t \right) \right]$$

$+A_{2}\left[ erfexp(F,t_{0},\tau_{\infty}=\infty,t) \right]+A_{3}\left[ erfexpRise(F,t_{0},\tau_{\mathrm{elastic}},t) \right]$ (S4)

where

$\mathrm{erfexpRise}\left( F,t_{0},\tau_{n},t \right)=erfexp\left( F,t_{0},\tau_{\infty}=\infty,t \right)-erfexp(F,t_{0},\tau_{n},t)$ (S5)

The oscillation is fit in the first component which contains an amplitude *A*_1_, a damping term *τ*_damp_, oscillation period *T*, and oscillation phase *φ*. The erfexp is the function resulting from the convolution of an exponentially decaying step function with a Gaussian. The second component accounts for the magnitude of the signal step jump, *A*_2_. For the third component, we add an exponential rise function, which is needed in the high power scans to account for a cooperative HS amplification effect.^[13]^ The *F* is the full-width at half maximum of the instrument response (IRF), *t*_0_ is time zero, and *τ*_elastic_ is the elastic timescale. For batch **2** (which is low power), *A*_3_ was held at 0. The oscillation constants were selected as local parameters for each particle size to produce a good fit. The measurements were performed from pre-time zero to 45 picoseconds after time zero. This was sufficient to interrogate the relevant dynamics without involving population decay.

**S5.3. Fitting Results**

The parameters *F*, *t*_0_, and *τ*_elastic_ were fixed from results of the solvent IRF analysis and high power fitting. The fitted parameters are shown in **Table S5**.

**Table S5.** The results of the size-dependent oscillation global fit. The standard deviations from the fit results are given.

|  | ***T* (ps)** | ***τ*_damp_ (ps)** | ***φ* (radians)** | **\|*A*_1_/*A*_2_\|** |
| --- | --- | --- | --- | --- |
| Small (**1**) | 2.3 ± 0.3 | 0.60 ± 0.08 | -0.5 ± 0.3 | 0.18 ± 0.02 |
| Medium (**2**) | 4.2 ± 3 | 0.7 ± 0.2 | 0.2 ± 1 | 0.55 ± 0.33 |
| Medium (**2***) | 5.5 ± 0.4 | 0.90 ± 0.06 | 0.89 ± 0.07 | 0.30 ± 0.05 |
| Large (**3**) | 14.8 ± 18 | 1.3 ± 0.3 | 1.1 ± 0.6 | - 1. ± 0.59 |

**S5.4. Estimate of Speed of Sound and Estimate of Period of Extensional Mode**

The calculation of the speed of sound using the extensional mode hypothesis is unit conversion and calculation from the slope in Figure 3c. The speed of sound can be calculated from

$c_{\text{ext}}=\frac{2L}{T_{\mathrm{ext}}^{0}}$ (S6)

And since the slope in Figure 3c gives *T*_ext_/*L*, we simply take the reciprocal, multiply it by 2 and convert it to m/s. This gives the value of 14000 ± 2000 m/s (error is the standard deviation from the slope fit).

In the case of the breathing mode, the calculation is more difficult due to the dependence of the *τ* parameter on the Poisson ratio *ν*. Our approach was to use a conservative range of *ν* (0.3-0.4) and determine the range of $T_{\mathrm{br}}^{0}$ values. Therefore, we must determine *τ* for this range. And to do that, for each value of *ν*, we must minimize *τ* in the following equation:

$\tau J_{0}(\tau)=\frac{1-2\nu}{1-\nu}J_{1}(\tau)$ (S7)

we plotted the following function in Mathematica:

$y(\tau)=\left( \frac{1-2\nu}{1-\nu} \right)\frac{J_{1}(\tau)}{J_{0}(\tau)}-\tau$ (S8)

and found the smallest *x*-intercept (ignoring the trivial *x* = 0) for varying values of *ν*. The following Mathematica code will show the plot for *ν* = 0.34:

Plot[((1-2*0.34)/(1-0.34))*BesselJ[1, x]/BesselJ[0, x]-x, {x,0, 5}]

yielding the following graph (**Figure S26**) with *x*-intercept (i.e. *τ* value) of 2.174:


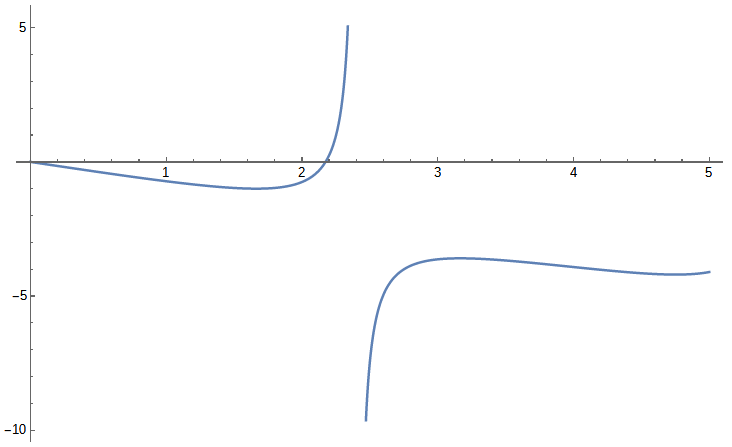


**Figure S26.** Mathematica plot of *τ* minimization at 0.34 *ν* value.

Using this procedure, we have determined *τ* for various values of *ν*, shown in **Table S6**. Once *τ* is determined, *c*_br_ can be calculated. We also have included the standard deviation from the error of the slope from the period versus width fit.

**Table S6.** The values of *τ*, *c*_br_, and it’s slope fit standard deviation *σ_c_*_,ext_ for various guess values of *ν*. The range of *c*_br_ is shown from the addition or subtraction of the slope fit error.

| ***ν*** | ***τ*** | ***c*_br_ (m/s)** | ***σ_c_*_,ext_ (m/s)** | ***c*_br_ ± *σ_c_*_,ext_ (m/s)** |
| --- | --- | --- | --- | --- |
| 0.25 | 2.069 | 1740 | 290 |  |
| 0.3 | 2.126 | 1600 | 270 | 2030 |
| 0.34 | 2.174 | 1460 | 250 |  |
| 0.35 | 2.187 | 1420 | 240 |  |
| 0.4 | 2.253 | 1200 | 200 | 990 |
| 0.45 | 2.326 | 870 | 150 |  |

Finally, we have added the variance of the range of Poisson ratios (0.3-0.4) to the standard deviation from the fit. This gives us the conservative range of 990 to 2030 m/s. We also note the value for Poisson ratio of 0.34 is 1460 m/s (since this was an experimentally relevant value for the Poisson ratio^[14]^).

**S5.5. Estimate of Extensional Mode Damping Time**

We do not observe the lower frequency *extensional* mode. This is due to a relatively short damping time compared to the oscillation period. If we only consider solely the damping of the oscillation due to nanoparticle size dispersion, a Gaussian distribution of nanorod lengths will give the following extensional mode damping time,^[15]^ *τ*_ext,damp_:

$\tau_{ext,damp}=\frac{\bar{L}\bar{T}}{\sqrt{2}\pi\sigma}$ (S9)

where $\bar{L}$ is the mean length, $\bar{T}$ is the mean period, and *σ* is the standard deviation of the nanoparticle distribution. The resulting *τ*_ext,damp_ gives an upper bound on the oscillation damping time. Using this relation—with the $\bar{L}$ and *σ* from TEM size analysis, we can calculate the upper bound damping times for our four batches (**Table S7**).

**Table S7.** Estimated extensional and breathing mode damping times from considering size dispersion. The observed damping time is shown in comparison. We also show an estimated extensional mode period.

|  | ***τ*_ext,damp_ (ps)** | ***τ*_br,damp_ (ps)** | ***τ*_obs,damp_ (ps)** | $\boldsymbol{T}_{\mathbf{ext}}^{\boldsymbol{0}}$ **(ps)** |
| --- | --- | --- | --- | --- |
| Small (**1**) | 2.2 ± 0.2 | 1.3 ± 0.1 | 0.60 ± 0.08 | 14.2 ± 0.2 |
| Medium (**2**) | 4.3 ± 2.1 | 1.9 ± 0.9 | 0.7 ± 0.2 | 38.9 ± 0.3 |
| Medium (**2***) | 9.9 ± 0.6 | 4.4 ± 0.3 | 0.90 ± 0.06 | 38 ± 2 |
| Large (**3**) | 13.2 ± 9.3 | 8 ± 6 | 1.3 ± 0.3 | 150 ± 10 |

Table S7 displays this *τ*_ext,damp_ calculation, the same calculated damping time for the breathing mode (*τ*_br,damp_), the experimentally determined damping time (*τ*_obs,damp_), and an estimated period of the fundamental extensional mode ($T_{\mathrm{ext}}^{0}$) assuming a speed of sound of 2000 m/s. Therefore, even with a conservative estimate of *τ*_ext,damp_, we expect the extensional mode signal would be washed out before a single beat.

**S6. Global Analysis (GA)**

**S6.1. Two-Exponential GA**

We performed a biexponential global analysis for all four batches. We used the following fit function:

$\Delta A\left( t \right)=A_{1}\mathrm{erfexp}\left( F,t_{0},\tau_{1},t \right)+A_{2}\mathrm{erfexp}\left( F,t_{0},\tau_{2},t \right)$ (S10)

The values of *F* and *t*_0_ were fixed from a solvent IRF analysis. The *τ* parameters were globally fit, and the amplitudes locally fit. The wavelength dependent amplitudes are the respective decay associated difference spectra (DADS) for the two exponential decay components (DADS_1_ and DADS_2_). The DADS are shown in **Figure S27-S30** for batches **1**, **2**, **2***, and **3**.


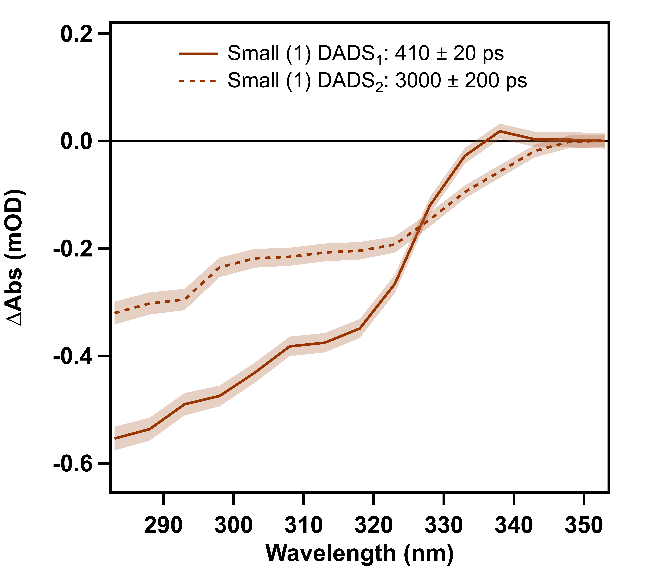


**Figure S27.** DADS for 2-state global analysis of batch **1**.


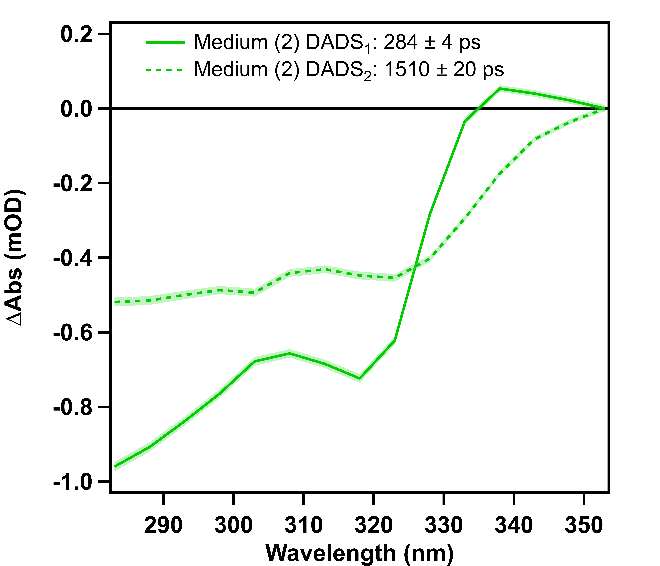


**Figure S28.** DADS for 2-state global analysis of batch **2**.


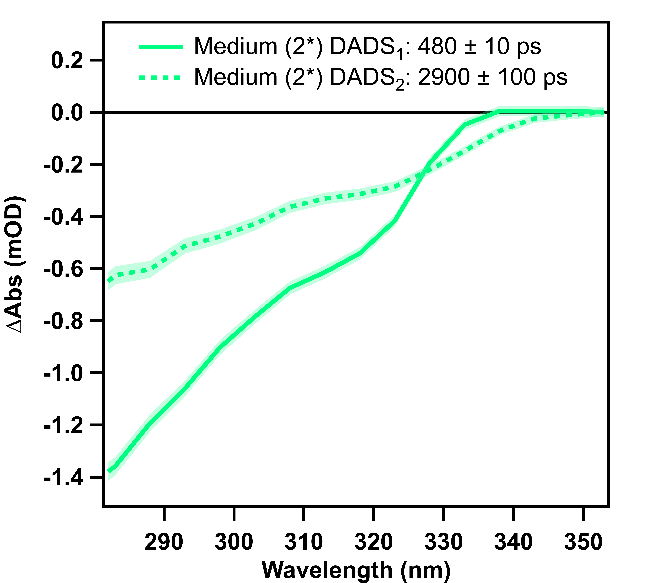


**Figure S29.** DADS for 2-state global analysis of batch **2***.


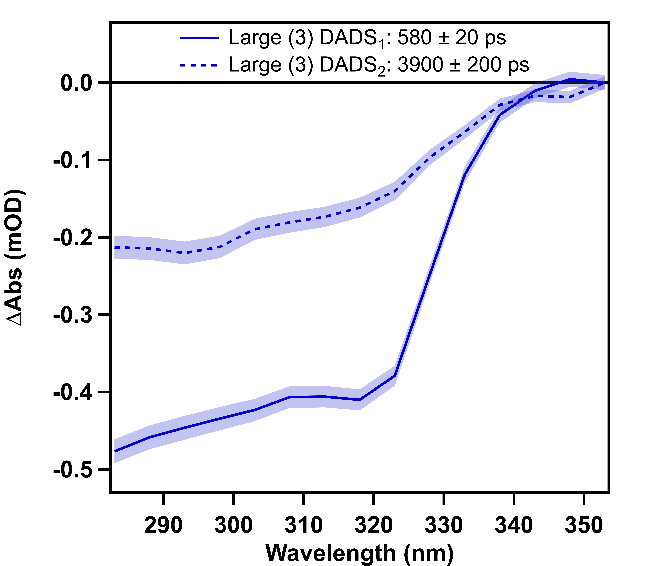


**Figure S30.** DADS for 2-state global analysis of batch **3**.

**S6.2. Three-exponential GA**

We performed a biexponential global analysis for all four batches. We used the following fit function:

$\Delta A\left( t \right)=A_{1}\mathrm{erfexp}\left( F,t_{0},\tau_{1},t \right)+A_{2}\mathrm{erfexp}\left( F,t_{0},\tau_{2},t \right)+A_{3}\mathrm{erfexp}\left( F,t_{0},\tau_{3},t \right)$ (S11)

Once more, the values of *F* and *t*_0_ were fixed from a solvent IRF analysis, the *τ* parameters were globally fit, and the amplitudes locally fit. The DADS are shown in **Figure S31-S34** for batches **1**, **2**, **2***, and **3**.


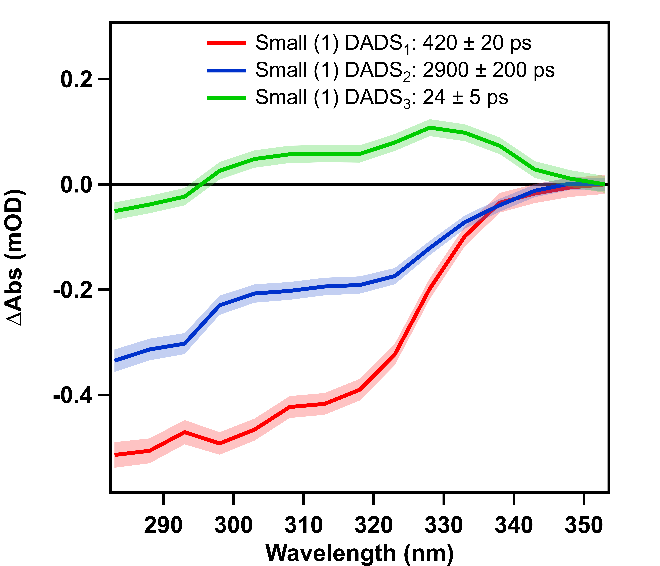


**Figure S31.** DADS for 3-state global analysis of batch **1**.


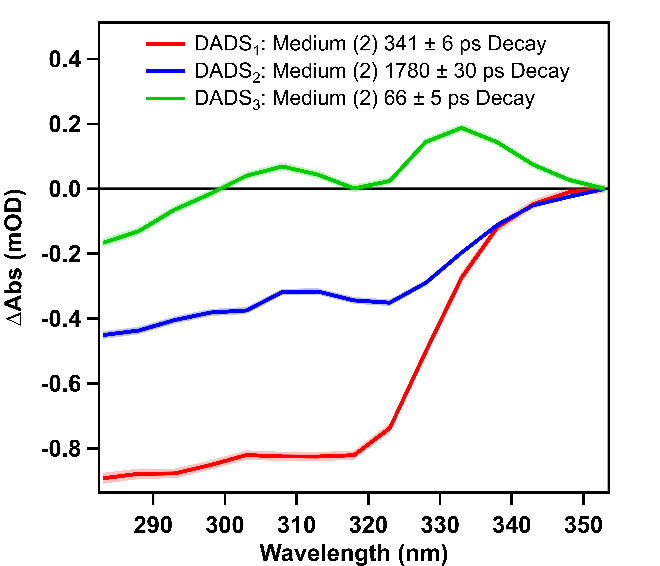


**Figure S32.** DADS for 3-state global analysis of batch **2**.


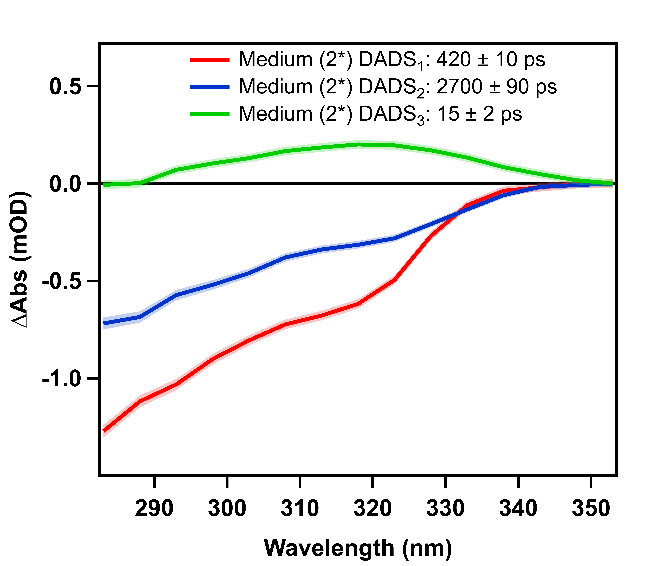


**Figure S33.** DADS for 3-state global analysis of batch **2***.


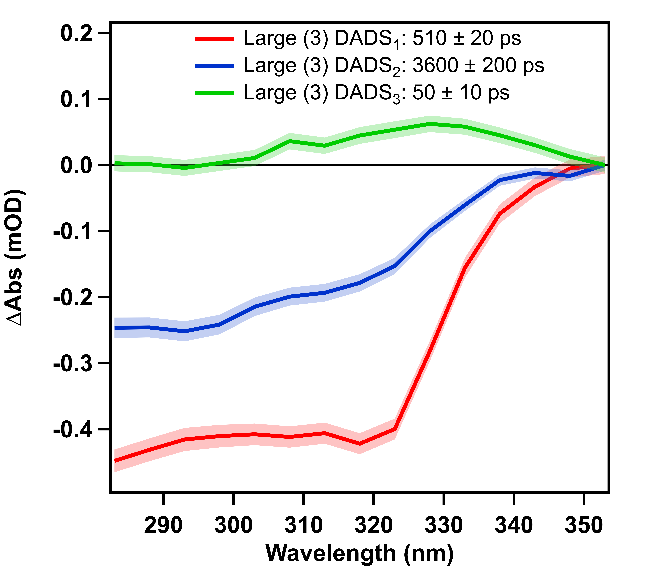


**Figure S34.** DADS for 3-state global analysis of batch **3**.

The improvement when adding the third exponential is significant in the 300-340 nm region, where DADS_3_ has the largest amplitude. This can be observed by comparing kinetic traces at select wavelengths to the 2 and 3-state global analyses in **Figure S35-S38**.


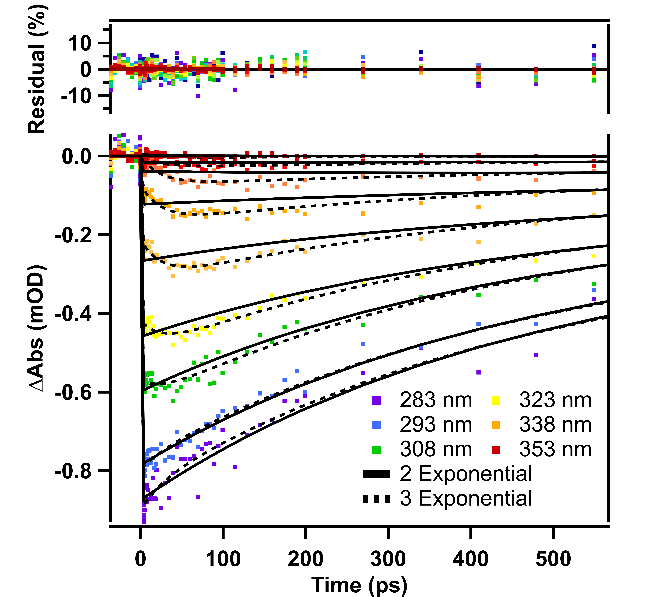


**Figure S35.** Comparison between 2 and 3-state global analysis for batch **1**.


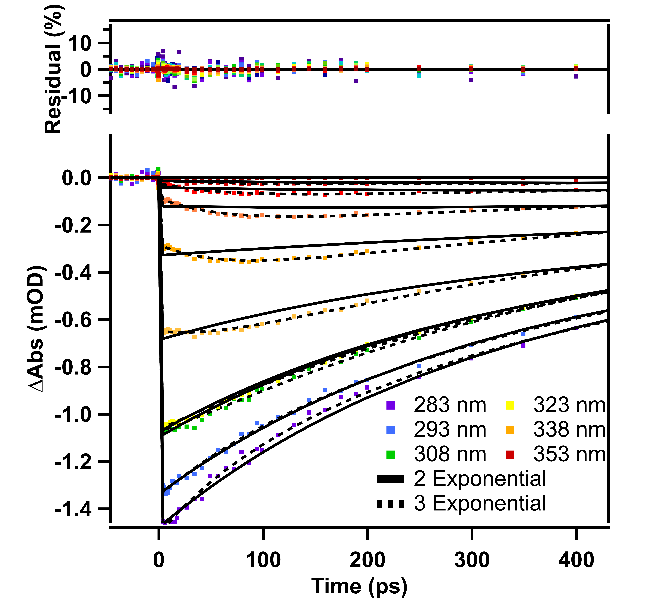


**Figure S36.** Comparison between 2 and 3-state global analysis for batch **2**.


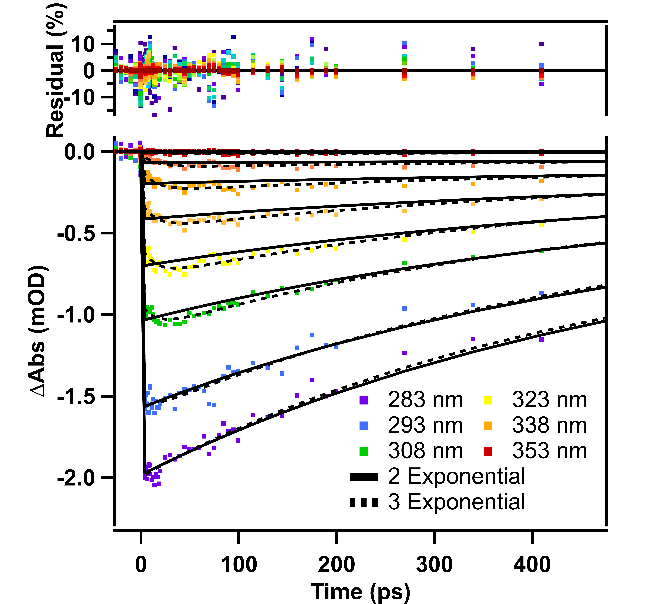


**Figure S37.** Comparison between 2 and 3-state global analysis for batch **2***.


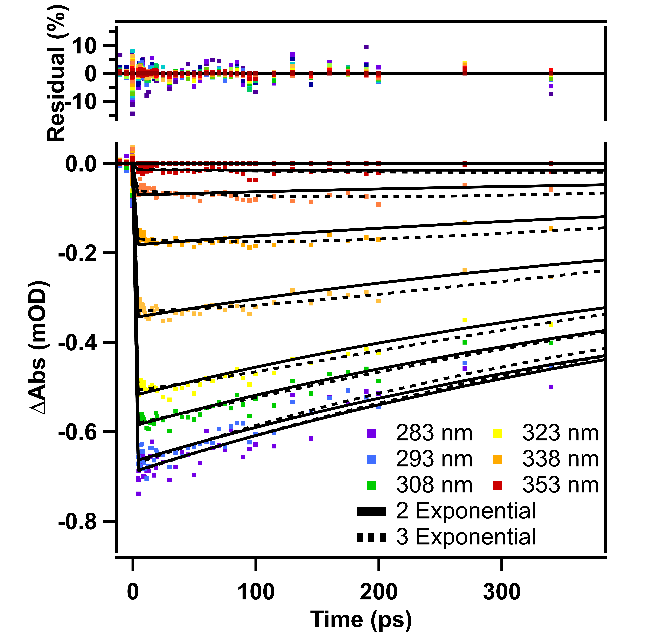


**Figure S38.** Comparison between 2 and 3-state global analysis for batch **3**.

**S6.3. Comparison of Exponential with Stretched Exponential and Distribution Fits**

We tested several different fit functions to model the ground state recovery. We fit a 280 nm kinetic trace of batch 2 from 50 ps to 7 ns using the exponential function ($y=A_{1}e^{-k_{1}t}$), biexponential function ($y=A_{1}e^{-k_{1}t}+A_{2}e^{-k_{2}t}$), and stretched exponential function ($y=A_{1}e^{-{(k_{1}t)}^{\beta}}$). We also performed fits using a normal and Log-normal distribution of rate constants.

**Figure S39** shows the fit results. The sum of squares of the residuals (RSS) was smallest for the double exponential function. Using Bayesian Information Criteria (discussed below), the stretched exponential function had a better BIC parameter and the double exponential function was second best. Given the more intuitive content in the exponential parameters, we decided to use the biexponential function for our analysis.


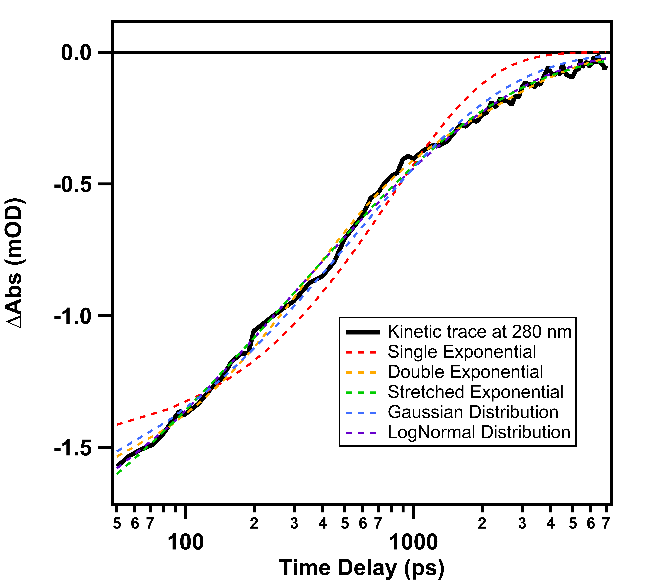


**Figure S39.** Modelling the ground state recovery using different fit functions.

**S7. Mechano-Elastic Monte Carlo Simulations**

We used the mechano-elastic model to simulate the non-equilibrium HS population dynamics in Fe-trz. Similar models have been previously used to simulate SCO dynamics after photoexcitation.^[13,16–18]^ Here, a 2D lattice is constructed of spheres to represent individual SCO centers. The spheres are positioned within a rectangular (to simulate a nanorod), open boundary layer with a triangular molecular geometry (**Figure S40**). The HS and LS states differ by their radius, with the HS having a larger size to match the experimental volume increase. The molecules are linked together by springs (of spring constant *k*) to represent the elastic interactions and introduce cooperativity to the system.


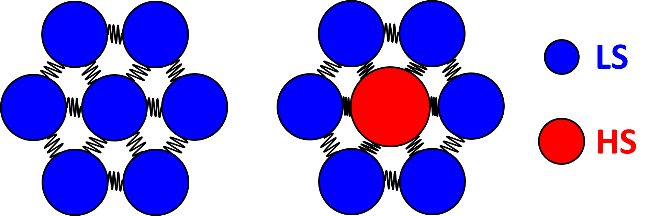


**Figure S40.** Ball and spring interactions in mechanoelastic model.

When—for any reason—a molecule flips its spin, its spherical area will change instantly, which causes a compression or elongation of the first coordination sphere of springs. This newly introduced strain will influence the position of molecules and cause a corresponding propagation of the local perturbation across the entire lattice.

**S7.1. Details about Simulations**

The Classical Hamiltonian for the mechanoelastic model is^[19]^

$\hat{H}=\frac{1}{2}\sum_{i} (D-k_{B}T\ln g)\sigma_{i}+\frac{k}{2}\sum_{i,j} \delta x_{i,j}^{2}$ (S12)

where the first sum is the Ising-like Gibbs free energy of the lattice and the second term is the total elastic energy from the sum of all spring interactions. Term one contains an enthalpy term (*D*) and the entropy difference between the spin states (*k*_B_*T* ln *g*). Here, the entropy variation is determined from the vibronic degeneracy ratio (*g*). The *σ_i_* parameter take the value of -1 for LS and +1 for HS.

The relaxation of the system is governed by Monte Carlo Arrhenius dynamics, where the transition probabilities are modulated by the activation energy (*E*_a_) barrier of the HS→LS transition. With this method, the transition probabilities (*P*) are^[20]^

$P_{\mathrm{HS}\longrightarrow\mathrm{LS}}^{i}=\frac{1}{\tau}\exp\left( -\frac{E_{a}+\kappa p_{i}}{k_{B}T} \right)$ (S13)

$P_{\mathrm{LS}\longrightarrow\mathrm{HS}}^{i}=\frac{1}{\tau}\exp\left( -\frac{E_{a}+\kappa p_{i}}{k_{B}T} \right)\exp\left( -\frac{D-k_{B}T\ln g}{k_{B}T} \right)$ (S14)

where *τ* is a constant which is scaled such that the probabilities much smaller than unity for all temperatures. The change in energy (for the *i*th molecule of local pressure, *p_i_*) is quantified by *κ*. The pressure is calculated from the sum of all neighboring spring interactions:

$p_{i}=\sum_{neighbors} k\delta x_{ij}$ (S15)

in which *δx_ij_* is a negative value for elongated springs and positive for compressed springs.

The advancement of the simulation occurs in steps—called a Monte Carlo step (MCS)—where in each step the switching probability for every molecule in the system is computed and compared with a random number, *η* ∈ [0,1]. If the transition probability is higher than *η*, then the transition is accepted and that molecule changes spin states; otherwise, that molecule remains in the same state. At the end of a MCS, the instantaneous radius change stemming from the molecules that change spin requires positions of molecules to be updated. This is done by solving a system of differential equations which account for the forces from the displacement of the springs for all molecules in the system:^[21]^

$\left\{ \begin{aligned} m\frac{d^{2}x_{i}}{dt^{2}}=F_{x}^{i}-\mu\frac{dx_{i}}{dt} \\ m\frac{d^{2}y_{i}}{dt^{2}}=F_{y}^{i}-\mu\frac{dy_{i}}{dt} \end{aligned} \right.$ (S16)

where *m* is the mass of each molecule, *x_i_*, *y_i_* are the coordinates of the molecules, $F_{x}^{i}$, $F_{y}^{i}$ are the vector components of the elastic force acting on each molecule, and *μ* is the linear damping constant.

The system is solved for a limited number of steps to simulate the non-equilibrium relaxation dynamics present in ultrafast laser experiments. The ratio *r* = *n*_1_/*n*_2_, where *n*_1_ is the number of steps in solving the differential equations, while *n*_2_ is the number of MCS between successive differential equation solutions.^[17]^ We set *r* at 2 for our ultrafast simulations.

The simulation starts with a pre-defined number of randomly chosen LS molecules to switch to HS, simulating a homogeneous photoexcitation. The laser-induced heating is accounted for by increasing the photoexcited molecules temperature by 850 K. The particle then is allowed to evolve by alternating between MCS computation and solving the differential equations at an *r* of 2. For plots of the relative HS populations (core, edge, and total) versus MCS, 4 sets of kinetic traces were averaged to produce the results in Figure 5a and 5c.

**S7.2. Determination of the Spring Constant**

Before simulating the phase transition curves, we allow the system to relax to the steady state position, where the net force acting on each molecule is zero. The thermodynamic parameters are fixed from previous DSC studies:^[22]^ ∆*H* = 3251 K and ∆*S* = 9.06 J/K (*g* = 8604). This gives rise to a transition temperature (*T*_C_ = ∆*H*/*k*_B_ ln *g*) of 358.8 K. The radius of HS molecules was set at 0.22 nm, while the LS radii were set to 0.20 nm. The *κ* parameter was fixed at 1450 K/N.^[20,23]^ The distance between the centers of neighboring molecules is 1 nm in LS state and 1.04 nm in HS state. The simulations were performed on rectangular-shaped systems of 10368 molecules with an aspect ratio of 4.


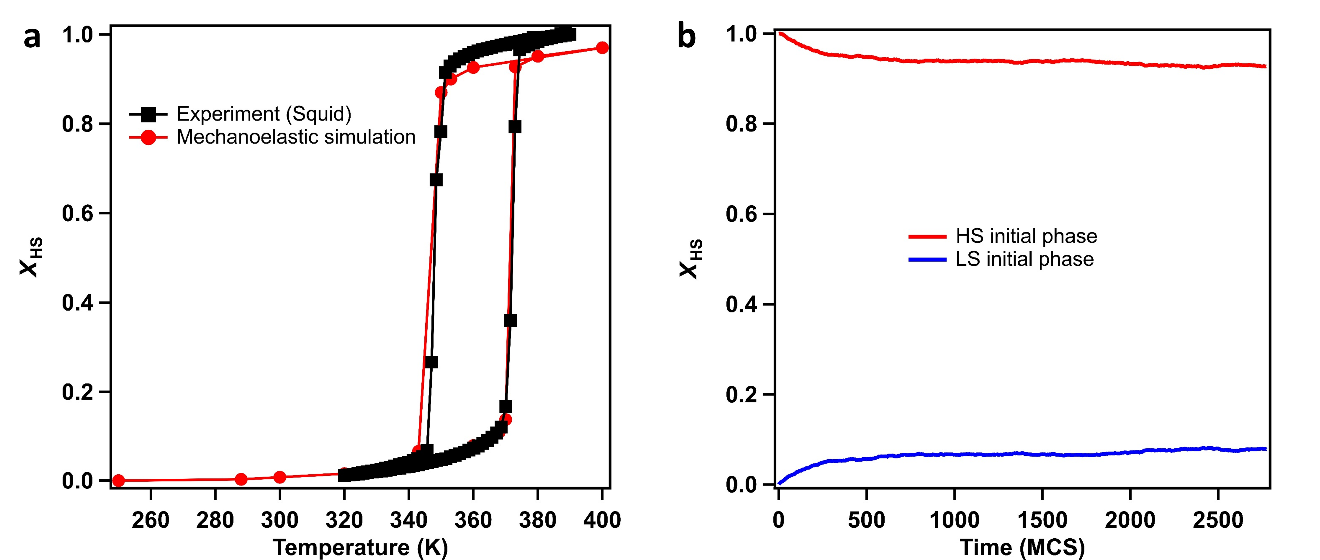


**Figure S41.** a) Simulated and measured LS↔HS phase transition curves. b) Relaxation of initially 100% LS and 100% HS lattices at 360 K.

With these parameters fixed a priori, the only variable which tunes the hysteresis magnitude spring constant *k*. We have plotted the experimental and simulated phase transition curves to determine the best value of *k* (**Figure S41**a)*.* In order to compare the experiment and simulation, we scaled the SQUID data from batch **2** to *Χ*_HS_ = 0 at 320 K and *Χ*_HS_ = 1 at 390 K. We obtained the best fit for *k* = 11 N/m. We computed relaxation curves from purely LS and HS lattices at 360 K (Figure S41b) in order to eliminate kinetic effects of ramp rate and minimizing the size effects.^[24]^ **Figure S42** shows snap-shots of steady-state particles at various temperatures. The proportion of HS molecules towards the edge is larger as a result of minimization of the elastic energy.^[23,25]^


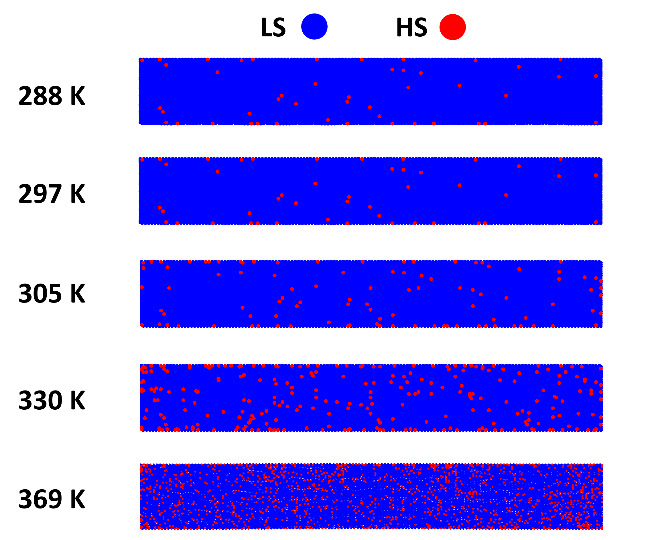


**Figure S42.** Snap-shots of steady state particles simulated at different temperatures. The HS circles were enlarged for the 4 lowest temperatures to increase visibility.

**S7.3. Heat Diffusion and Temperature Dynamics in the Core, Edge, and Entire Particle**

After photoexcitation—where the photon energy exceeds the HS - LS energy difference—the excess energy is transferred to the lattice. A study using ultrafast time-resolved mid-IR absorption experiments estimated that the heat transfer occurred with *τ* ≈ 6 ps for a particular SCO complex.^[26]^ With this timescale in mind, we must consider the dynamic nature of the temperature within Fe-trz. In our simulations, the temperature of each (*i*th) molecule is determined using the following heat diffusion equation:^[18]^

$\frac{dT^{i}}{dt}=-\alpha\cdot(T^{i}-\left\langle T_{ij} \right\rangle)-\beta\cdot(T^{i}-T_{B})$ (S17)

where *α* is the internal heat diffusion coefficient, *T^i^* is the temperature each molecule, ⟨*T_ij_*⟩ is the average temperature of all neighbors (*j*) of each molecule, *β* is the heat diffusion coefficient to the bath (considered only for edge molecules), and *T*_B_ is the thermal bath temperature (288 K). We used thermal coefficients of *α* = 10^-2^MCS^-1^ and *β* = 10^-2^MCS^-1^, which reproduced our experimental kinetics.

**S7.3. Simulation with Reduced Thermal Diffusion**

To test whether the simulated amplification of HS population at the particle edges is a result of diffusion of heat (e.g. from the photoexcited molecules to their neighbors), we have performed MC simulations with 100 times lower heat diffusion constants (*α* = 10^-4^MCS^-1^ and *β* = 10^-4^MCS^-1^). **Figure S43** shows the results from non-equilibrium MC simulations for an initial photoexcited HS population of 2% with reduced heat diffusion. We plot the simulated dynamics of the edge and core HS fractions separately.


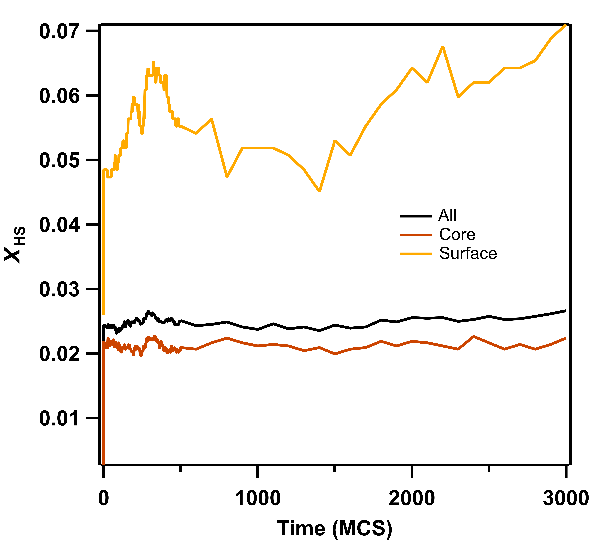


**Figure S43.** Results from MC simulations with 100-fold smaller heat diffusion coefficients. The total (all) HS, edge HS, and core HS fractions are plotted as a function of time (MCS). Fractions are relative to the respective number of Fe centers at the edge (887) and in the core (9481).

As with Figure 5c, we observe a HS amplification of the edge particles. We do not see a simultaneous *(de)amplification* of core HS species, likely due to the reduced heat diffusion. The photoexcited molecules in the core remain hot much longer. Therefore, the counteracting large positive pressure (which favors HS→LS) and large temperature (which favors LS→HS) will allow the particles to remain HS for longer, until the molecules start to dissipate heat at much longer time intervals.

**S8. Target Fit Analysis**

**S8.1. Details of the Fit Function and Definition of Parameters**

The simultaneous population loss of core species with increase of surface species was modeled in a target fit using the following equation:

$$\Delta A\left( t \right)=A_{C}\mathrm{erfexp}\left( F,t_{0},\tau_{C},t \right)$$

$$+A_{S}\mathrm{erfexp}\left( F,t_{0},\tau_{S},t \right)+x_{C}A_{C}\mathrm{erfexp}\left( F,t_{0},\tau_{C,el},t \right)$$

$+x_{S}A_{S}\frac{\tau_{S}}{\tau_{S}-\tau_{S,el}}[erfexp\left( F,t_{0},\tau_{S,el},t \right)-erfexp\left( F,t_{0},\tau_{S},t \right)]$ (S18)

The first and second terms represent the equivalent terms as DADS_1_ and DADS_2_ from the 3-state global analysis. Namely, they account for the decay of the photoexcited core (*A*_C_) and surface (*A*_S_) species with their respective decay constants *τ*_C_ and *τ*_S_.

Term 3 gives the loss of HS population of core particles due to the elastic effect (de-amplification) with time constant *τ*_C,el_. The amplitude of this de-amplification is represented as a *fraction* *x*_C_ of *A*_C_. Therefore, while *A*_C_ is a wavelength dependent parameter which scales based on the wavelength dependent species profile, *x*_C_ is a single value representing a scaling factor for the *A*_C_ profile.

Term 4 represents the increase in HS population of surface particles to elastic amplification with time constant $\tau_{C,el}$. The amplitude is again represented as a fraction *x*_S_ of the wavelength dependent amplitude profile of the surface species *A*_S_. This increased population ultimately decays with the surface decay constant $\tau_{S}$.

**Figure S44** shows the target analysis fit compared with selected kinetic traces for all 4 batches.


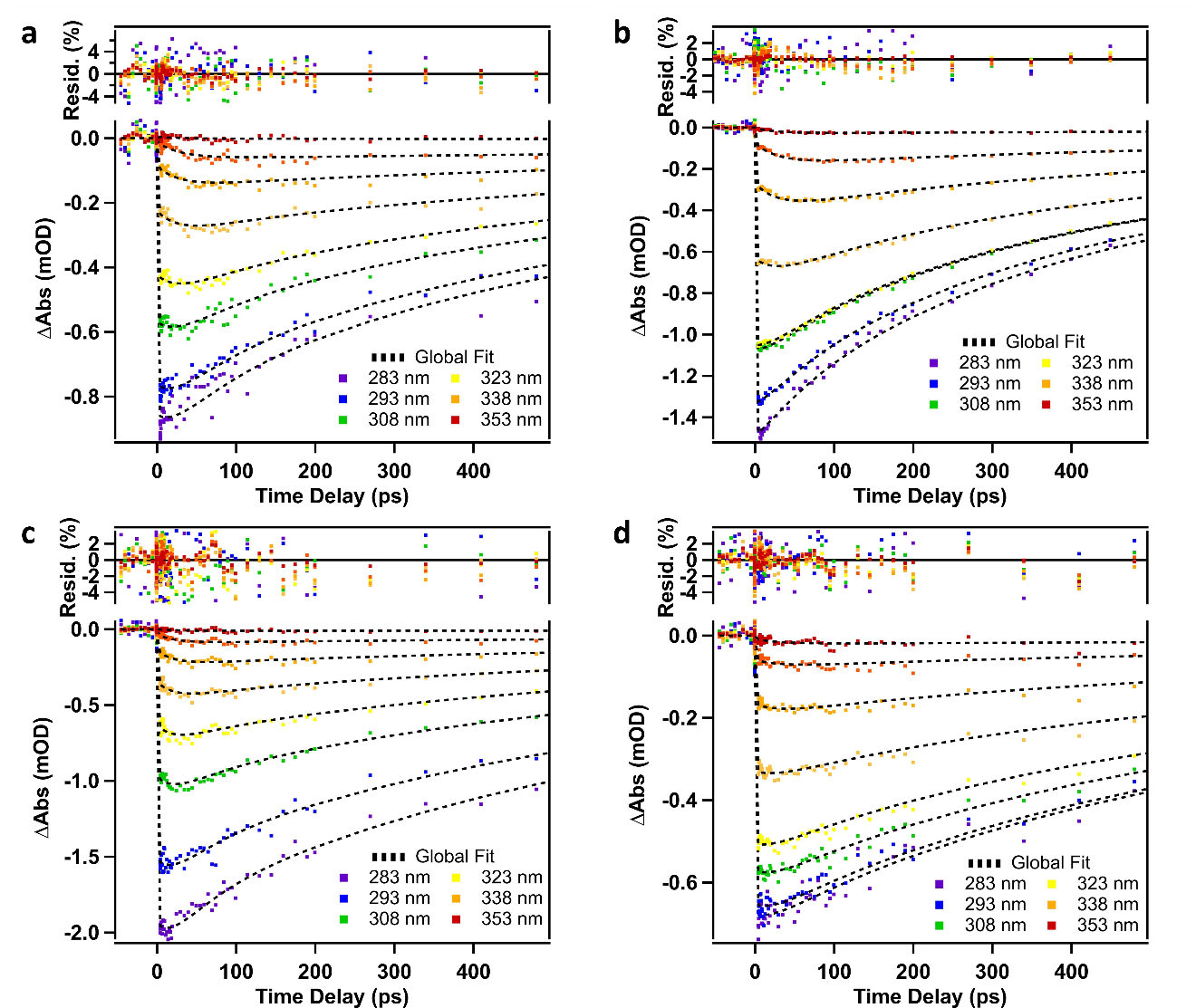


**Figure S44.** Target analysis using Equation S18. Kinetic traces at selected wavelengths compared with the global fit curves. The residuals are shown above. a) Batch **1**. b) Batch **2**. c) Batch **2***. d) Batch **3**.

**Figure S45** shows the amplitude profiles (SADS) of the core and surface species (which are difference spectra). The products *x*_C_*A*_C_ and *x*_S_*A*_S_ are also shown. The SADS profiles were fit with a two Gaussian model.


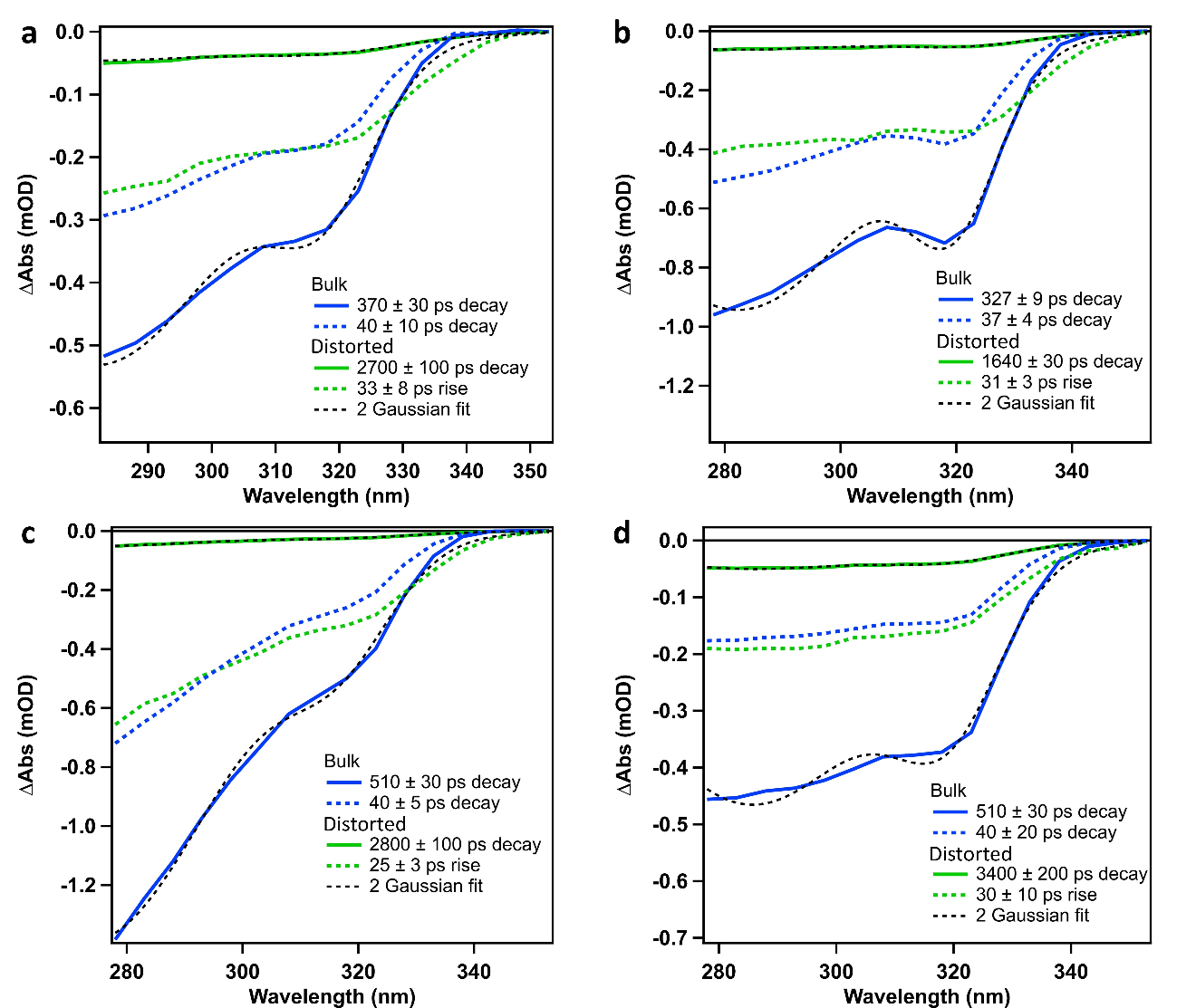


**Figure S45.** Target analysis using Equation S18. The SADS_1_ and SADS_2_ and extracted time constats are shown. a) Batch **1**. b) Batch **2**. c) Batch **2***. d) Batch **3**.

**S8.2. Comparison in Fit Quality between Target Fit and Three-Exponential Fit**

In the analysis of transient absorption data, the researcher must balance fidelity with complexity in the fitting model. Fidelity can be achieved when the fit produces well-behaving residuals and a sufficiently small residual sum of squares, RSS:

$RSS=\sum_{i=1}^{n} \left( O_{i}-f_{i} \right)^{2}$ (S19)

where *O_i_* is the *i*th experimental value, *f_i_* is the *i*th fitted value, and *n* is the number of data points. A model should also be minimally complex yet yield meaningful parameters.

Many models will adequately reproduce the experimental data and so we may choose the best ones using criteria which consider *χ*^2^ and quantify the complexity. The reduced chi-squared value, $\chi_{\nu}^{2}$ = RSS/*ν*, to some extant accounts for the complexity with the degree of freedom, *ν* = *n* – *m*, where *m* is the number of parameters. Minimizing $\chi_{\nu}^{2}$ is how many modelling software packages determine the final fit parameters. In broadband transient absorption, there are often thousands or 10’s of thousands of data points in a single wavelength:time image plot. Such a large *n* means that the magnitude of *ν* will be less affected by *m*. Therefore, $\chi_{\nu}^{2}$ becomes insensitive to addition of more parameters.

Other measures of model quality exist which have a more severe consequence of increasing *m*. In order to compare our target model and 3 exponential global analysis, we have evaluated the results using the Akaike information criteria (AIC) and Bayesian information criteria (BIC):

$AIC=n\ln\left( \frac{\mathrm{RSS}}{n} \right)+2m$ (S20)

$BIC=n\ln\left( \frac{\mathrm{RSS}}{n} \right)+m\ln n$ (S21)

These two criteria have been used in a handful of studies to evaluate ultrafast spectroscopy models.^[27,28]^ In **Table S8**, we show the comparison between the target analysis and global analysis using the discussed information criteria. Blue indicates a better performance and red a poorer one.

**Table S8.** Summary of the statistical comparison between the 3-state global analysis and target analysis for the 4 batches.

|  | **Model** | ***n*** | ***m*** | $\mathbf{RSS}$ | $\boldsymbol{\chi}_{\boldsymbol{\nu}}^{\mathbf{2}}$ | **AIC** | **BIC** |
| --- | --- | --- | --- | --- | --- | --- | --- |
| Small (**1**) | Target Analysis | 1650 | 37 | 9.7651 x 10-7 | 6.0540 x 10-10 | -34985 | -34785 |
|  | Global Analysis |  | 49 | 9.2653 x 10-7 | 5.7872 x 10-10 | -35048 | -34782 |
| Medium (**2**) | Target Analysis | 2265 | 37 | 3.3730 x 10-7 | 1.5139 x 10-10 | -51178 | -50966 |
|  | Global Analysis |  | 49 | 3.1801 x 10-7 | 1.4351 x 10-10 | -51287 | -51006 |
| Medium (**2***) | Target Analysis | 1725 | 37 | 1.8178 x 10-6 | 1.0769 x 10-9 | -35583 | -35381 |
|  | Global Analysis |  | 49 | 1.8258 x 10-6 | 1.0894 x 10-9 | -35552 | -35284 |
| Large (**3**) | Target Analysis | 1650 | 37 | 6.1622 x 10-7 | 3.8203 x 10-10 | -35747 | -35552 |
|  | Global Analysis |  | 49 | 5.8434 x 10-7 | 3.6499 x 10-10 | -35798 | -35506 |

The more parameter-punishing BIC favors the target model with fewer parameters in all batches except **2** (where BIC is very close). Remarkably, for batch **2*** even the RSS is lower for the target analysis, despite having fewer parameters. Therefore, we find a favorable statistical justification for using a physically relevant kinetic model over a non-specific global analysis with additional parameters.

**S8.3. Spectral Fitting and Extraction of Surface and Core Spectra**

The results of the 2 Gaussian fitting of the SADS are shown in **Table S9**.

**Table S9.** The results of the fit using Equation S2 on the SADS_1_ and SADS_2_ of batches **1**, **2**, **2***, and **3**.

|  |  | ***μ*_1_ (nm)** | ***σ*_1_ (nm)** | ***A*_2_/*A*_1_** | ***μ*_2_ (nm)** | ***σ*_2_ (nm)** |
| --- | --- | --- | --- | --- | --- | --- |
| Small (**1**) | Core | 281.6 ± 1 | 21.5 ± 2 | 0.33 ± 0.11 | 317.7 ± 0.4 | 8.0 ± 0.8 |
|  | Surface |  |  | 0.13 ± 0.03 | 319.0 ± 4 | 12.8 ± 4 |
| Medium (**2**) | Core | 282.3 ± 1 | 23.1 ± 2 | 0.16 ± 0.004 | 319.8 ± 0.3 | 7.6 ± 0.5 |
|  | Surface |  |  | 0.31 ± 0.06 | 322.0 ± 5 | 12.0 ± 5 |
| Medium (**2***) | Core | 273.0 ± 3 | 23.4 ± 3 | 0.08 ± 0.01 | 316.8 ± 0.7 | 9.1 ± 1 |
|  | Surface |  |  | 0.18 ± 0.1 | 317.9 ± 10 | 12.7 ± 10 |
| Large (**3**) | Core | 285.7 ± 0.9 | 22.0 ± 2 | 0.20 ± 0.01 | 319.4 ± 0.4 | 8.9 ± 0.9 |
|  | Surface |  |  | 0.24 ± 0.04 | 320.2 ± 4 | 10.8 ± 4 |

All batches show a red-shifted and broadened ^1^T_2g_ band in the surface species (compared to the core). All batches (except **1**) show a more intense ^1^T_2g_ band in the surface species. To show the reconstructed absorption spectra of the surface and core species, we have reproduced the results of the above Gaussian fit for batch **2** in Figure 7.

**S8.4. Size Dependence from Target Fit**

From the target fit, we can compare the size-dependence of the core and surface species fractions. For each batch, we determined the core “population” *P*_C_ signal by summing the *A*_C_ profile from 283-318 nm (inclusive) and subtracting the *x*_C_*A*_C_ profile. This wavelength range was used to avoid the geometry-dependent spectral differences between the ^1^A_1g_→^1^T_2g_ GSB feature. Similarly, the surface population *P*_S_ was determined by summing the *A*_S_ profile from 283-318 nm (inclusive) and adding the *x*_S_*A*_S_ profile. The *P*_C_ and *P*_S_ values are dependent on the transient GSB signal amplitudes. However, the relative fractions *P*_C_/(*P*_C_ + *P*_S_) and *P*_S_/(*P*_C_ + *P*_S_) are representative of the relative population fractions since the GSB is proportional to ΔHS. These fractions and the standard deviation from error propagation were determined for all 4 batches and are given in Table 1 of the main text.

**S9. Density Functional Theory Calculations**

**S9.1. Details of the Calculations**

We performed a density functional theory (DFT) optimization of the Fe-trz molecular analogue, bis[hydrotris(1,2,4-triazol-1-yl)borate]iron(II) complex ([Fe(HB(tz)_3_)_2_]) using the crystal structure of the LS phase reported elsewhere.^[29]^ The Hessian matrix was created by calculating the second derivatives of energy along the nuclear coordinates. The eigenvalues of the Hessian are the frequencies, and the eigenvectors are the normal modes. The existence of all positive-valued eigenvalues confirmed that the structure was at a minimum on the potential energy surface. The Orca program was used to calculate the oscillator strengths of the ^1^MLCT and d-d electronic transitions at varied ground state geometries. The TPSSh functional and def2-svp basis set were used in each calculation.

**S9.2. Oscillator Strength as a Function of Geometric Distortion**

To examine how the oscillator strength of Fe-trz’s electronic transitions are influenced by geometric distortions (expected for surface states), we calculated the oscillator strength between the ground and the excited states along a normal mode which breaks the symmetry. In this mode, 3 Fe-N distances are extended and the other 3 distances are contracted (**Figure S46**). Small distortions significantly increase the oscillator strength of the d-d transitions (while having a small effect on the ^1^MLCT states), consistent with our experimental results.


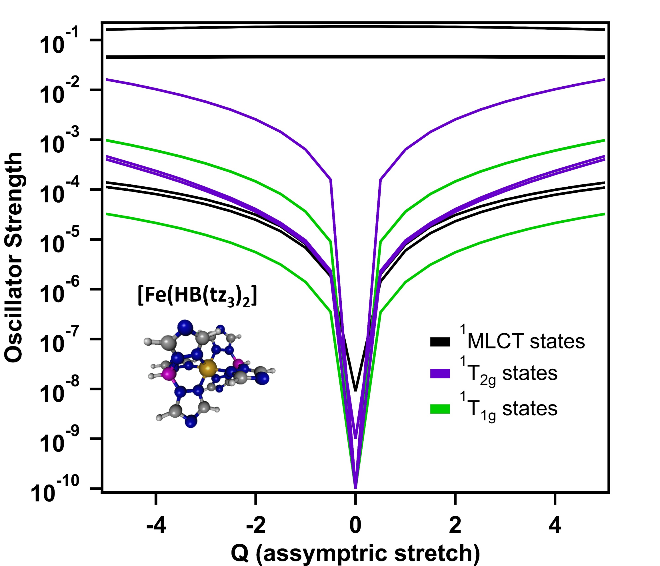


**Figure S46.** The oscillator strength of electronic transitions in [Fe(HB(tz)_3_)_2_] as a function of displacement magnitude along a symmetry breaking mode.

**S9.3. DFT Short-Chain Simulations**

To demonstrate the increased stabilization of the HS state at the surface (terminus) for an Fe-trz-like chain complex, we extended the [Fe(HB(tz)_3_)_2_] analogue to a 4 Fe atom chain. Here, one side represents a surface geometry in which each triazole ligand has an uncoordinated nitrogen bond to terminate the chain. To perform these calculations, we optimized the geometry (at the aforementioned DFT level) for the HS center in the different Fe sites: 1 to 4 (**Figure S47**). In position 1, the HS is at the “surface.” Positions 2 to 4 represent the core, with 4 having the HS at the opposite Fe center (near the BH group). The final geometries with the HS on the different Fe atoms can be obtained by extending the Fe-N distances about each center. Then, we produced the different geometries (HS-LS-LS-LS, LS-HS-LS-LS, LS-LS-HS-LS and LS-LS-LS-HS, for the 4 different Fe centers 1-2-3-4). After obtaining the different geometries, we generated the intermediate geometries using the Geodesic program, enabling the geometries to be connected through a minimum energy path. Then, we performed DFT electronic structure calculations at those different geometries. We used the different wavefunctions in the sequential geometries to calculate the energy difference between the four different HS positions. The crossing points represent the energy at cost to transfer a HS from one Fe atom to another.


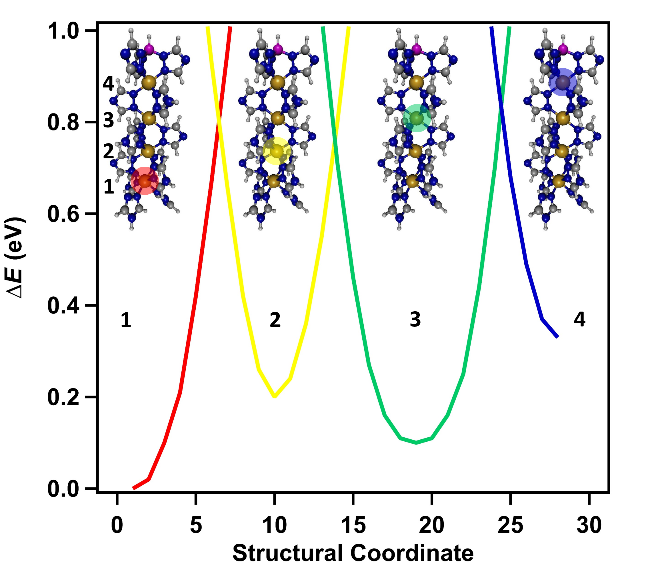


**Figure S47.** The relative energy of different HS geometry positions for a 4 Fe Fe-trz molecular analogue.

**S10. Thermogravimetric Analysis**

A Q50 thermogravimetric analyzer (TGA) was used to verify the removal of NaAOT surfactant after washing. Using the degradation from 310 to 400 °C as an Fe-trz mass marker, we calculate a mass loss of 1.099% for unwashed and 49.54% for washed Fe-trz. Therefore, we can estimate 49.54/1.099 = 45.077 times more Fe-trz in the washed sample than the unwashed by mass. From the synthesis starting materials (20 g NaAOT, 1 g Fe precursor, and 0.6 g triazole precursor) the initial fraction of NaAOT is 20/(21.6) = 92.6% by mass (assuming unity synthetic yield). After washing, NaAOT reduces to less than 2.1% (92.6/45.077) by mass.


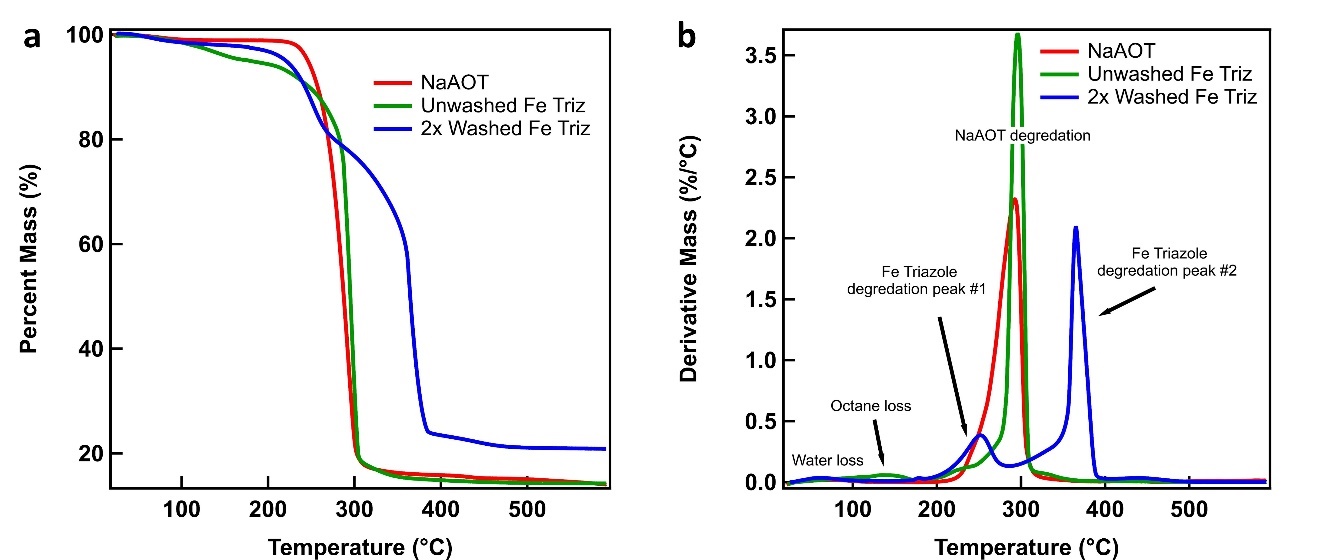


**Figure S48.** a) The percent mass loss as a function of temperature for NaAOT, unwashed Fe-trz, and washed Fe-trz. b) The derivative of plot. Arrows point to particular mass loss events

References

[1] E. Coronado, J. R. Galán-Mascarós, M. Monrabal-Capilla, J. García-Martínez, P. Pardo-Ibáñez, *Adv. Mater.* **2007**, *19*, 1359.

[2] J. R. Galán-Mascarós, E. Coronado, A. Forment-Aliaga, M. Monrabal-Capilla, E. Pinilla-Cienfuegos, M. Ceolin, Inorg*. Chem.* **2010**, *49*, 5706.

[3] C. Bartual-Murgui, E. Natividad, O. Roubeau, *J. Mater. Chem. C* **2015**, *3*, 7916.

[4] M. Giménez-Marqués, M. L. García-Sanz De Larrea, E. Coronado, *J. Mater. Chem. C* **2015**, *3*, 7946.

[5] A. Grosjean, P. Négrier, P. Bordet, C. Etrillard, D. Mondieig, S. Pechev, E. Lebraud, J. F. Létard, P. Guionneau, *Eur. J. Inorg. Chem.* **2013**, 796.

[6] G. A. Bain, J. F. Berry, *J. Chem. Educ.* **2008**, *85*, 532.

[7] S. Brooker, *Chem. Soc. Rev.* **2015**, *44*, 2880.

[8] P. J. M. Johnson, V. I. Prokhorenko, R. J. D. Miller, *Opt. Express* **2009**, *17*, 21488.

[9] K. Zhang, M. F. Lin, E. S. Ryland, M. A. Verkamp, K. Benke, F. M. F. De Groot, G. S. Girolami, J. Vura-Weis, *J. Phys. Chem. Lett.* **2016**, *7*, 3383.

[10] K. Zhang, R. Ash, G. S. Girolami, J. Vura-Weis, *J. Am. Chem. Soc.* **2019**, *141*, 17180.

[11] Y. Shari ati, J. Vura-Weis, *J. Synchrotron Radiat.* **2021**, *28*, 1850.

[12] R. Ash, *PhD Thesis*, University of Illinois Urbana-Champaign, November, **2020**.

[13] R. Bertoni, M. Lorenc, H. Cailleau, A. Tissot, J. Laisney, M. L. Boillot, L. Stoleriu, A. Stancu, C. Enachescu, E. Collet, *Nat. Mater.* **2016**, *15*, 606.

[14] M. Mikolasek, M. D. Manrique-Juarez, H. J. Shepherd, K. Ridier, S. Rat, V. Shalabaeva, A. C. Bas, I. E. Collings, F. Mathieu, J. Cacheux, T. Leichle, L. Nicu, W. Nicolazzi, L. Salmon, G. Molnár, A. Bousseksou, *J. Am. Chem. Soc.* **2018**, *140*, 8970.

[15] M. Hu, X. Wang, G. V Hartland, P. Mulvaney, J. P. Juste, J. E. Sader, *J. Am. Chem. Soc.* **2003**, *125*, 14925.

[16] R. Bertoni, E. Collet, H. Cailleau, M.-L. L. Boillot, A. Tissot, J. Laisney, C. Enachescu, M. Lorenc, *Phys. Chem. Chem. Phys.* **2019**, *21*, 6606.

[17] C. Enachescu, L. Stoleriu, M. Nishino, S. Miyashita, A. Stancu, M. Lorenc, R. Bertoni, H. Cailleau, E. Collet, *Phys. Rev. B* **2017**, *95*, 224107.

[18] Y. Hu, M. Picher, N. M. Tran, M. Palluel, L. Stoleriu, N. Daro, S. Mornet, C. Enachescu, E. Freysz, F. Banhart, G. Chastanet, *Adv. Mater.* **2021**, *33*, 2105586.

[19] C. Enachescu, M. Nishino, S. Miyashita, L. Stoleriu, A. Stancu, *Phys. Rev. B: Condens. Matter Mater. Phys.* **2012**, *86*, 54114.

[20] C. Enachescu, A. Hauser, *Phys. Chem. Chem. Phys.* **2016**, *18*, 20591.

[21] C. Enachescu, L. Stoleriu, A. Stancu, A. Hauser, *Phys. Rev. Lett.* **2009**, *102*, 1.

[22] K. Jonas, A. Jean-Paul, C. Renée, C. Epiphane, K. Olivier, J. G. Haasnoot, G. Françoise, J. Charlotte, A. Bousseksou, L. Jorge, V. François, G. V. Anne, *Chem. Mater.* **1994**, *6*, 1404.

[23] C. Enachescu, M. Nishino, S. Miyashita, K. Boukheddaden, F. Varret, P. A. Rikvold, *Phys. Rev. B: Condens. Matter Mater. Phys.* **2015**, *91*, 104102.

[24] L. Stoleriu, P. Chakraborty, A. Hauser, A. Stancu, C. Enachescu, *Phys. Rev. B: Condens. Matter Mater. Phys.* **2011**, *84*, 1.

[25] A. I. Popa, L. Stoleriu, C. Enachescu, *J. Appl. Phys.* **2021**, *129*, 131101.

[26] A. Volte, C. Mariette, R. Bertoni, M. Cammarata, X. Dong, E. Trzop, H. Cailleau, E. Collet, M. Levantino, M. Wulff, J. Kubicki, F. L. Yang, M. L. Boillot, B. Corraze, L. Stoleriu, C. Enachescu, M. Lorenc, *Commun. Phys.* **2022**, *5*, 1.

[27] C. Ruckebusch, M. Sliwa, P. Pernot, A. de Juan, R. Tauler, *J. Photochem. Photobiol., C* **2012**, *13*, 1.

[28] E. Wang, K. S. Specht, A. J. Chicco, J. W. Wilson, *J. Phys. Chem. B* **2022**, *126*, 1404.

[29] S. Rat, K. Ridier, L. Vendier, G. Molnár, L. Salmon, A. Bousseksou, *CrystEngComm* **2017**, *19*, 3271.
